# Virtual Screening and Network Pharmacology-Based Study to Explore the Pharmacological Mechanism of *Clerodendrum* Species for Anticancer Treatment

**DOI:** 10.1155/2022/3106363

**Published:** 2022-11-02

**Authors:** Barbi Gogoi, S. P. Saikia

**Affiliations:** ^1^Agrotechnology and Rural Development Division (ARDD), CSIR-North East Institute of Science and Technology, Jorhat 785006, Assam, India; ^2^Academy of Scientific and Innovative Research (AcSIR), Ghaziabad 201002, India

## Abstract

**Background:**

Cancer is a second leading cause of death in the world, killing approximately 3500 per million people each year. Therefore, the drugs with multitarget pharmacology based on biological networks are crucial to investigate the molecular mechanisms of cancer drugs and repurpose the existing drugs to reduce adverse effects. *Clerodendrum* is a diversified genus with a wide range of economic and pharmacological properties. Limited studies were conducted on the genus's putative anticancer properties and the mechanisms of action based on biological networks remains unknown. This study was aimed to construct the possible compound/target/pathway biological networks for anticancer effect of *Clerodendrum* sp. using docking weighted network pharmacological approach and to investigate its potential mechanism of action.

**Methods:**

A total of 194 natural *Clerodendrum* sp. Compounds were retrieved from public databases and screened using eight molecular descriptors. The cancer-associated gene targets were retrieved from databases and the function of the target genes with related pathways were examined. Cytoscape v3.7.2 was used to build three major networks: compound-target network, target-target pathway network, and compound-target-pathway network.

**Results:**

Our finding indicates that the anticancer activity of *Clerodendrum* sp. involves 6 compounds, 9 targets, and 63 signaling pathways, resulting in multicompounds, multitargets, and multipathways networks. Additionally, molecular dynamics (MD) simulations were used to estimate the binding affinity of the best hit protein-ligand complexes. Conclusion. This study suggests the potential anticancer activity of *Clerodendrum* sp. which could further contribute to scavenger novel compounds for the development of new alternative anticancer drugs.

## 1. Introduction

Cancer is a neoplasmic disease that regulates uncontrolled cell division and leads to abnormal growth of cell mass. In 2018, the World Cancer Report estimated about 1.16 million new cases with 784,800 deaths and 2.2.6 million in 5-year prevalent cases in India. The National Cancer Institute had listed eight categories for the cancer treatment which include chemotherapy, radiation, surgery, targeted therapy, stem cell transplant, and medicines and immunotherapy [1]. Some of these therapies have resulted in drug resistance after initial positive response which is termed as acquired drug resistance that occur in both cytotoxic chemotherapies and targeted therapies. Moreover, cancer involves the interactions of multiple genes as well as functional proteins. The success of drug discovery in cancer remains unsolved due to diversity of cancer types, excessive toxicity, constraints efficacy, and acquired treatment resistance [2]. This complication in cancer treatment arises due to the failure of “one gene, one drug, one disease” paradigm [3, 4]. Therefore, the developments of new potent and nontoxic treatments are highly active in scientific research [5].

With the development of computational approach, disease networks constructed through network biology are suggested as powerful tools for screening out drugs targets. Network pharmacology developed by Hopkins enhances high rate of clinical success with less side effects, and around 40% of the current drugs discoveries were contributed by this approach [3]. This approach is best appreciated in anticancer research, where both genetic and nongenetic bypass mechanism has led to inherent in different cancer phenotypes [6, 7].

The practices of natural products from plants are widely accepted as potential new lead source towards the discovery of new alternative anticancer drug. The secondary metabolites extracted from plants were considered as the primary source of drugs in Indian and other ancient systems of medicine in the world due to its higher structural diversity, metabolism in the body with low toxicity, and complexity than synthetic drugs [8]. These bioactive natural compounds could inhibit potential targets and reduce the cost of new drug development due to its availability in nature as well as provide option for combination therapies [9]. Since 1981 to 2014, a total of 174 compounds were approved for commercialization in the treatment of cancer. Many studies suggested that the modulation of multitargets rather than single targets could lead to discovering effective drugs [10]. Several studies reported the use of different plant-based resources for the treatment of cancer with scientific validations but a majority of plants were still left to be documented [11, 12]. The *Clerodendrum* sp. reported to inhibit cancer activity but its efficiency and mechanism remain unclear until today [13]. In this study, we focused on chemical constituents of *Clerodendrum* to be effective against cancer. The genus *Clerodendrum* consists of 580 species that are widely distributed in tropical and subtropical regions of the world that comprises of small trees, shrubs, and herbs [14]. Many researchers isolated and identified different biological active compounds and other major chemical constituents such as flavonoids, phenolics, steroids, and terpenoids from the genus [15–17]. More than 280 major chemical constituents from various species of *Clerodendrum* were isolated and identified till date. Many species of this genus were used as folk medicines by various tribes of African and Asian continents for the treatment of life-threatening diseases, anticancer, antitumor, antidiabetic, antihypertensive, and antidiarrhoel activities [18, 19]. Besides its medicinal importance, this genus has ornamental values. Species such as *C. thomsoniae*, *C. indicum*, *C. panniculatum*, *C. inerme*, *C. japonicum*, and *C. speciosum* are cultivated for their aesthetic values. *C. inerme*, *C. thomsonia,* and *C. splendens* are among the most sought for cultivation in gardens, for covering fences and walls.

**Figure 1:**
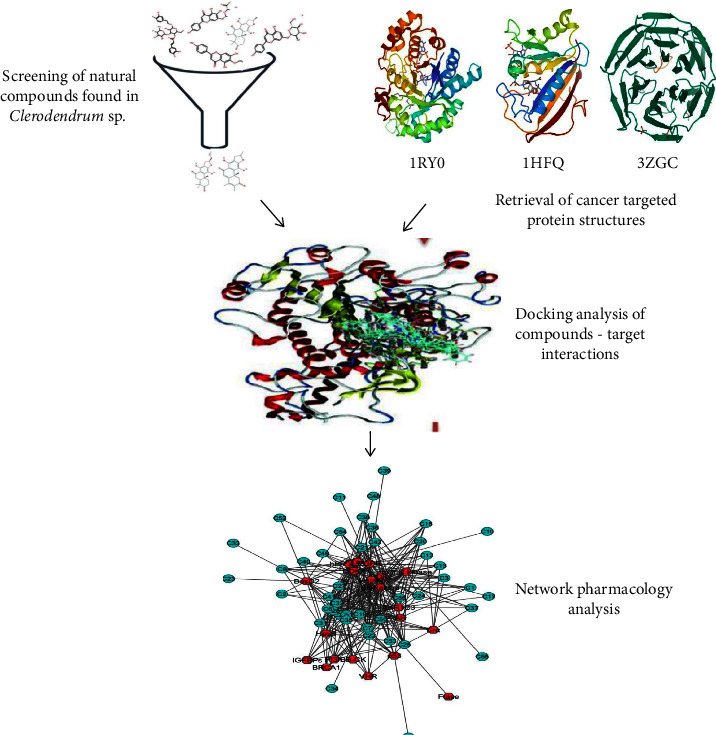
Graphical representation of this study.

Even though there are many studies on pharmacological effects of the genus but the anticancer activity of the genus based on in silico analysis and network pharmacology were elucidated till date. Therefore, this study was aimed to explore the active compounds, potential targets, and biological pathways of the *Clerodendrum* sp. using molecular docking, network pharmacology, and molecular simulations analysis, which could further provide basis for subsequent studies and clinical applications. The detail flowchart of this study was depicted in Figure 1.

## 2. Material and Methods

### 2.1. Collection of Natural Compounds and Space Analysis

The bioactive natural compounds (NCs) of *Clerodendrum* sp. were retrieved through extensive literature survey [20–23]. The two dimensional (2D) structures of the compounds were retrieved from PubChem and Chemspider databases and prepared into a library. The duplicate structures were deleted and some compounds which did not have specific structures were sketched using the Marvin Sketch v6.2.0 and saved in.mol2 format. The two dimensional (2D) structures were converted into 3D structures by using Corina 3D analysis tool in Tsar (Tools for structure activity relationships) software (https://www.accelrys.com/). Further, the three dimensional (3D) structures were converted to.pdb format. The dataset of compounds was further analysed, and hydrogen was added using CHARMm based on smart minimizer that generates 1500 steps of Steepest Descent followed by conjugate gradient algorithms with a convergence gradient of 0.001 kcal mol. The details of bioactive compounds retrieved from *Clerodendrum* sp. are listed in Table S1.

Additionally, 43 approved drugs molecules related to cancer were collected from Drug Bank and selected as reference to analyse the NCs library. The molecular descriptors of NCs and drugs were calculated using the PaDEL-Descriptor software.

The principal component analysis (PCA) was conducted on the NCs and drug library by using the BioVinci tools to visualize the distribution of libraries in the chemical space. The PCA analysis was performed with 8 molecular descriptors: ALogP, Molecular Weight, Number H-Donors, Number-H Acceptors, Number Rotatable Bonds, Number of Rings, Number of Aromatic Rings, and Molecular Fractional Polar Surface Area.

### 2.2. ADME and Toxicity Profiling

The ADMET study refers to the pharmacokinetics approach of a molecule where absorption, distribution, metabolism, excretion, and toxicity of the compounds could be analysed. The ADME and toxicity properties of the selected NCs were predicted using PreADMET server and Osiris Property Explorer (https://www.openmolecules.org/propertyexplorer/applet.html).

### 2.3. Retrieval of Cancer Targets

The protein targets associated with human cancer were collected from four resources: (i) Therapeutic Target Database (TTD) [24], (ii) DrugBank [25], (iii) Uniport [26], and (iv) Protein Data Bank [27]. The duplicates structures, structures with no active site, incomplete structures, and structures from other organisms were deleted. Finally, 60 important drug targets from key type ‘*Homo sapiens'* were selected. The information of the collected protein targets is listed in Table S2. The downloaded PDB structures were selected based on the parameters such as (a) Protein extracted from X-ray diffraction process and (b) must contain one or more active sites for binding of ligands. All the structures were cleaned and optimized using USCF Chimera [28]. Among 60 3D protein structures, the resolutions of crystal structures were found to be ranging from 1.2 Å to 3.5 Å.

### 2.4. Protein-Protein Interaction (PPI) Network Analysis

The PPI data was obtained from STRING 11.0 (Search Tool for the Retrieval of Interacting Genes/Proteins) with species restricted to “*Homo sapiens*.” This database constructs nodes and edges of network to represent proteins and protein-protein associations. To ensure high confidence information, the minimum score was set to >0.9 and excluded the disconnected proteins in the networks. The potential target proteins and protein pathways involved in cancer were recorded in an excel table and imported into the Cytoscapev3.7.2 to obtain target pathway network.

### 2.5. Gene Ontology (GO) and Koyo Encyclopedia of Genes and Genomes (KEGG) Pathway Analysis of Cancer Targets

All the potential target genes were uploaded to DAVID (Database for Annotation, Visualization and Integrated Discovery) database. The identifier was selected as ‘OFFICIAL GENE SYMBOL', and species were selected as ‘*Homo sapiens'* for GO (Gene Ontology) enrichment analysis and KEGG (Kyoto Encyclopaedia of Genes and Genomes) pathway annotation [29].

### 2.6. Molecular Docking Approach

The bioactive NCs were docked to target protein using PyRx tool [30]. In this study, AutoDockVina, which allows flexible docking in active site by allowing flexibility in the ligand, was used for docking [31]. Blind docking was performed in NCs against cancer targeted proteins in order to detect the possible binding sites and modes of the ligands by examining the complete mass of targeted protein. Based on docking score the results were analysed and ranked accordingly. Further, these docking results were subjected to the construction of biological networks to study the polypharmacological nature of selected NCs as cancer inhibitors.

### 2.7. Network Pharmacology Study

Network Pharmacology approach was used to predict the polypharmacological potency of NCs to identify potential anticancer drugs [32]. The three networks were constructed as follows: (i) compound-target network, (ii) target-target pathway network, and (iii) compound-target-pathway network for better understanding of the active compounds and their mechanism against cancer. All the networks were visualized with the Cytoscape v3.7.2 software and analysed using the Network Analyzer plugin [33].

### 2.8. Molecular Dynamics Simulation

MD simulation was conducted to analyse the stability of protein-ligand complexes between *Clerodendrum* NCs and potential cancer targets. In this study, NVIDIA RTX 1060 GPU accelerated GROMACS 2021 software; running over Linux Ubuntu 20.04 LTS operating system was used. The topology of protein and ligands were generated using Charmm36 force field and SwissPARAM server [34]. The solvation of each system was performed with TIP3P water model followed by neutralization with suitable Na^+^ and Cl^−^ ions. The energy was minimized by the steepest descent minimization algorithm with maximum 50,000 steps. Position restrains were applied to receptor and ligand of the each systems for 100 ps throughout heating (300 K) utilizing NVT (No. of atoms, Volume, Temperature) together with leap-frog integrator, 2 fs time step, and LINCS holonomic constraints. After NVT, NPT (No. of atoms, Pressure, Temperature) equilibration was conducted at 300K temperature for 100 ps using 2 fs time step. Finally, 10 ns MD production run was generated without any restrain with 2 fs time step, and structures were recorded at each 10 ps coordinates. The trajectories files of root mean square deviation (RMSD), root mean square fluctuation (RMSF), Radius of gyration (Rg), and a number of hydrogen bond analysis (H-bonds) were calculated after completion of MD simulations for further analysis.

## 3. Results

### 3.1. Screening of Active NCs of *Clerodendrum* sp

**Figure 2:**
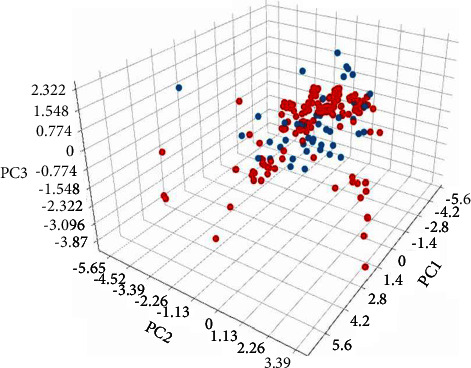
PCA space analysis between NCs and Drug Libraries. Red Circles and Blue circles represent NCs and Drugs, respectively.

A total of 194 NCs of *Clerodendrum* sp. were screened to create a compound library and evaluated with the eight molecular descriptors such as molecular weight (MW), number of hydrogen bond acceptors (HBAs), number of hydrogen bond donors (HBDs), total polar surface area (TPSA), and the drug-like properties of the NCs and drugs. In this study, it was found that out of 194, 126 natural products satisfied the eight physiological conditions and the details are provided in Table 1. A similar set of results were analysed for reported drug compounds. Further, a target library of 60 cancer proteins was compiled. The NCs of *Clerodendrum* and reported drug libraries were further evaluated using PCA to visualize its allocation in the chemical space. The eight molecular descriptors were used to generate the PCA model. We found that the distributions of NCs (Red sphere) are analogous to the 3D space occupied by the reported compounds (Blue sphere) and specify the resemblance of drug like assets in NCs with potential anticancer activity. The variances of PCA1, PCA2, and PCA3 are -1.98, -1.45, and -3.27, respectively (Figure 2).

The structures of 126 compounds were imported to PreADMET server and Osiris Property Explorer. The results of ADME/toxicity screening showed that 58 compounds had good ADME parameters and relatively safe to be considered as drug-like compounds for inhibition of cancer. Hence, the selected NCs satisfies the overall parameters to be taken over as positive drugs and listed in Table 2 where “Green” indicates drug-conform behaviour.

### 3.2. GO and KEGG Pathway Enrichment Analysis

The potential 60 cancer targets genes were uploaded to DAVID 6.8 database for GO annotation and KEGG pathway analysis. The threshold was set as *P* ≤ 0.05, and the pathways or gene function with maximum count were analysed further.

**Figure 3:**
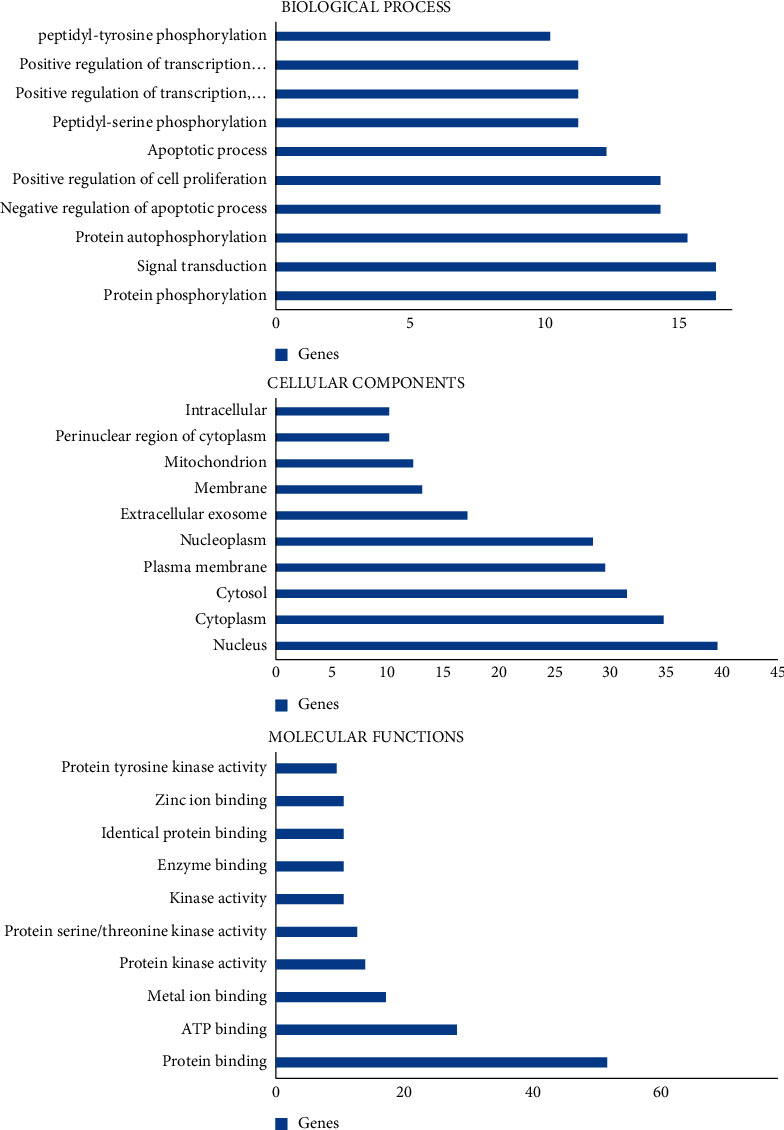
The top 10 GO terms of candidate cancer targets.

GO annotation analysis showed that the number of genes involved in CC, MF, and BP targets was 60 (Figure 3). CC enrichment recorded 29 chart records such as nucleus (39, 65.0%), cytoplasm (34, 56.7%), and cytosol (31, 51.7%). MF analysis recorded 45 chart records which were mainly involved in protein binding (49, 81.7%), ATP binding (27, 45.0%), metal ion binding (16, 26.7%), etc. Similarly, BP enrichment involved 161 chart records in the following target genes such as protein phosphorylation (16, 26.7%), signal transduction (16, 26.7%), and protein autophosphorylation (15, 25.0%).

**Figure 4:**
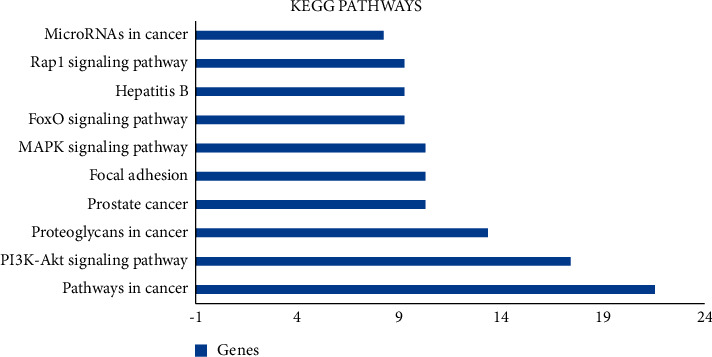
Top 10 components of KEGG pathway analysis.

KEGG pathway annotation showed that 54 out of 60 (90.0%) potential targets were enriched and involve 71 pathways, and 63 of these pathways were significantly correlated with target genes (*P* ≤ 0.05). The following pathways with largest number of genes involved were cancer pathways (22, 36.7%), PI3K-Akt signalling pathway (18, 30.0), proteoglycan in cancer (14, 23.3%), prostate cancer (11, 18.3%), Focal adhesion (11, 18.3%). The comparative analysis of 10 KEGG pathways with the strongest correlation revealed that among the 54 potential targets genes, the genes repeatedly associated with these pathways were MAPK1, PIK3CA, HRAS, EGFR, TP53, PRKCA, SRC, PDPK1, TGFBR2, TGFBR1, ERBB2, CASP3, BRCA2, XIAP, FGFR3, and CDK2 which are depicted in Figure 4.

### 3.3. Analysis of Target Proteins in Protein-Protein Interaction Network

**Figure 5:**
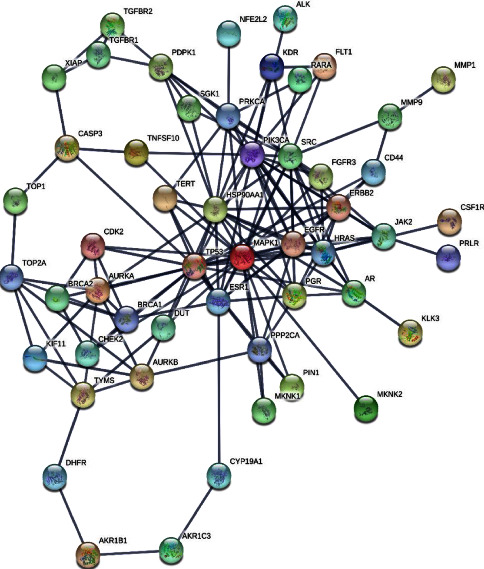
PPI network of cancer targeted proteins.

PPI network was constructed to analyse the physical interactions of 60 cancer proteins by using STRING software. The data were downloaded in CSV file format and imported into Cytoscape v3.7.2 software [10]. In PPI networks, a higher degree value of a node indicates the importance of node in the network. A total of 149 edges and 52 nodes with an average node degree of 4.97 were obtained the in PPI network after filtering the confidence level >0.9 and rejecting the target protein independent of the network. The network analyser tool indicates that the proteins MAPK1, TP53, HSP90AA1, SRC, PIK3CA, HRAS, EGFR, ESR1, ERBB2, PRKCA, AURKA, BRCA1, AR, PGR, JAK2, PDPK1, PPP2CA, TYMS, TOP2A, AURKB, CDK2, and TERT contained degree higher than average node degree, which means that these proteins might play an essential role of bridge to connect other nodes in PPI network (Figure 5).

### 3.4. Molecular Docking of NCs with Cancer Targets

**Figure 6:**
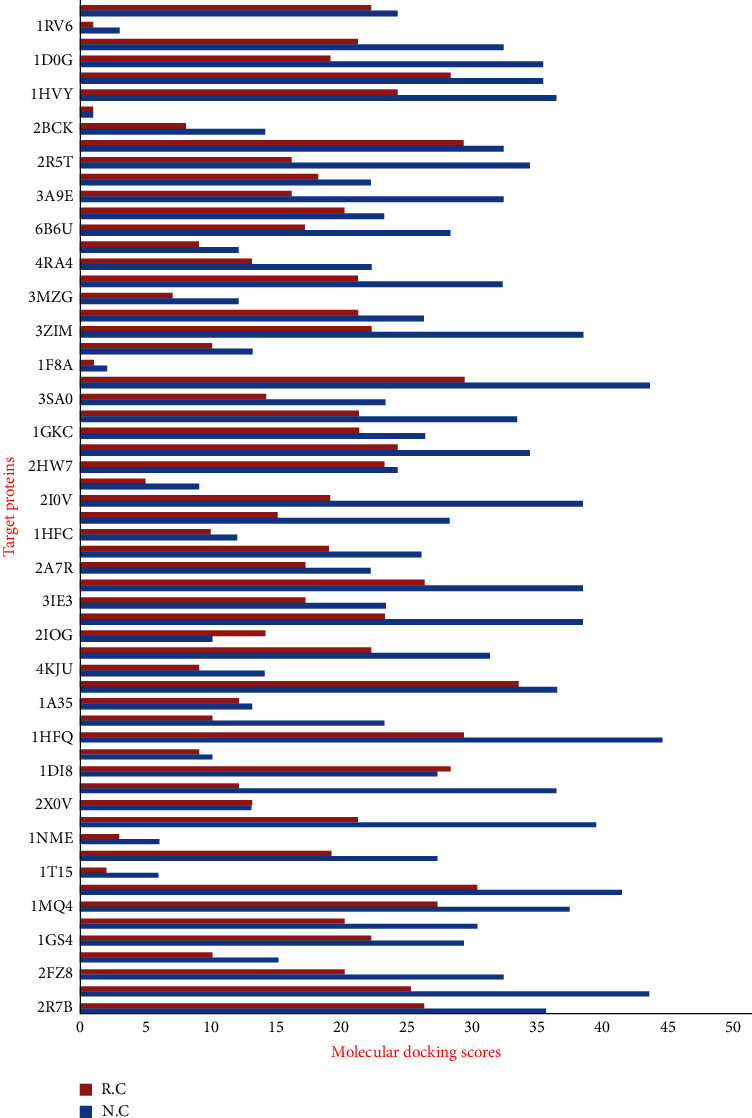
Compounds with docking score lower than −8.0 kcal/mol with cancer drug targets. In Figure ‘NC' and ‘RC' depicts natural compounds and reported compounds.

In this study, the 58 screened NCs were docked with 60 selected cancer target proteins using AutoDockVina in the PyRx tool. The NCs were considered as ligands against 60 targeted cancer genes. A similar method was applied for the reported drugs to have a comparative study. All the NCs were found to bind with 60 potential targets resulting in different docking scores. Based on the docking score a threshold value of lower than −8.0 kcal/mol was selected and ranked the compounds based on this threshold value (Figure 6). The cancer target PDB ID 1RY0 (AKR1C3) showed the highest interactions with the majority of the NCs, followed by 1HFQ (DHFR), 3ZGC (NFE2L2), 4AF3 (AURKB), 4K33 (FGFR3), 5P21 (HRAS), 2I0V (CSF1R), and 3ZIM (PIK3CA) protein targets and least interaction was found with the 1M9Z (TGFBR2) protein. Further, these docking results were subjected for the NCs-Cancer target network construction in order to study the polypharmacological nature of selected NCs as cancer inhibitors.

### 3.5. Construction of Biological Networks Using

#### 3.5.1. Compound-Target (C-T) Network Analysis

**Figure 7:**
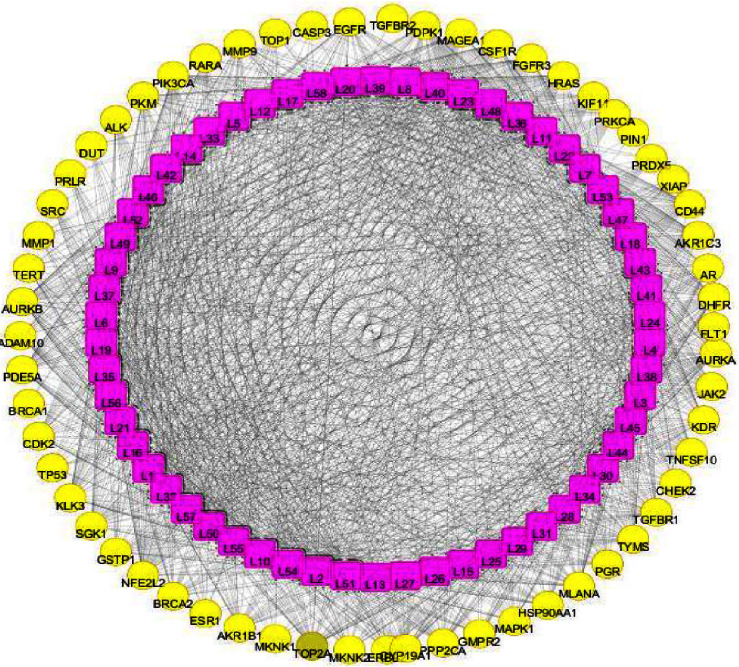
Compound–target network. Pink rectangle represents the compounds of *Clerodendrum* sp., yellow circles represent targets of cancer and edges represent interaction between ingredients and targets.

The C-T network was constructed with a docking score (lower than −8.0 kcal/mol) to understand the mechanism of action between NCs of *Clerodendrum* and cancer targets (Figure 7). The C-T network consists of 118 nodes (58 compounds and 60 potential targets) and 1492 edges (C-T interactions). The possible interaction between NCs and target proteins wasevaluated with important topological parameters such as degree, average betweenness centrality of nodes, closeness centrality, average shortest path length, and Topological Coefficient. We found that 57 compounds (expect L13) interact with multiple targets and 59 targets expect TGFBR2 interacts multiple compounds in the constructed network. Thus, these findings indicate multicomponents multitargets interaction in *Clerodendrum* sp.

#### 3.5.2. Target-Target Pathway Analysis (T-P)

**Figure 8:**
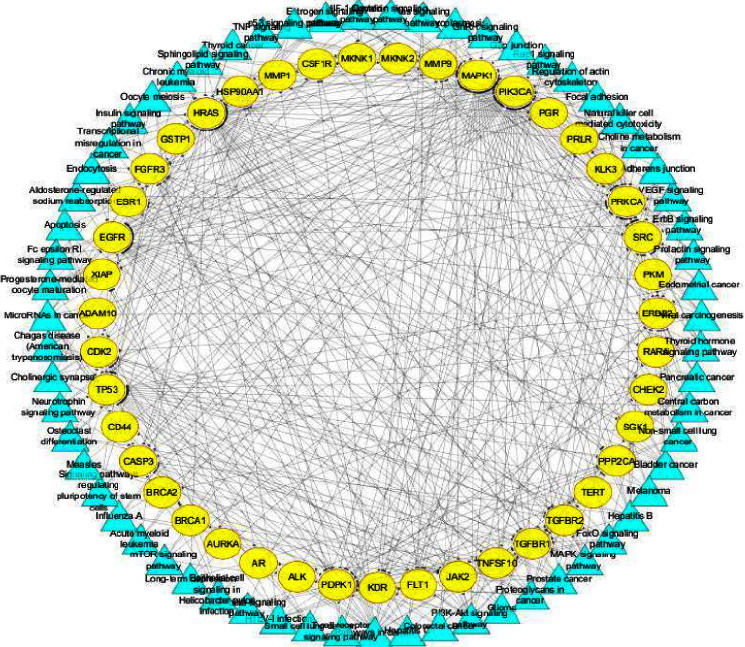
Target–target pathway network. Yellow circles represent targets of cancer and Blue triangle represents the pathways.

A total of 63 pathways related to cancer (*P* ≤ 0.05) were obtained by placing cancer-targeted genes in DAVID for KEGG pathway analysis. We constructed the T-P network by combining the pathways with cancer-related gene information. This network analyses the interaction of cancer targets and relationship between pathways and targets (Figure 8). The network consists of 106 nodes (63 pathways and 43 genes) and 435 edges. The results showed that pathway in cancer contained maximum genes (22°) followed by PI3K-Akt signaling pathway, proteoglycan in cancer, prostate cancer, and focal adhesion could play a crucial role in the treatment of cancer and regulates the complex biological and metabolic processes. These pathways could be suggested as potential signaling pathways that mediate the effects of *Clerodendrum* NPs against cancer. Moreover, the target genes such as MAPK1, PIK3CA, HRAS, EGFR, TP53, PRKCA, PDPK,1and SRC genes could act on more than 18 pathways. Interfering target genes of these pathways could be a potential strategy for the future treatment of cancer disease.

#### 3.5.3. Compound-Target-Pathway Network Analysis (C-T-P)

**Figure 9:**
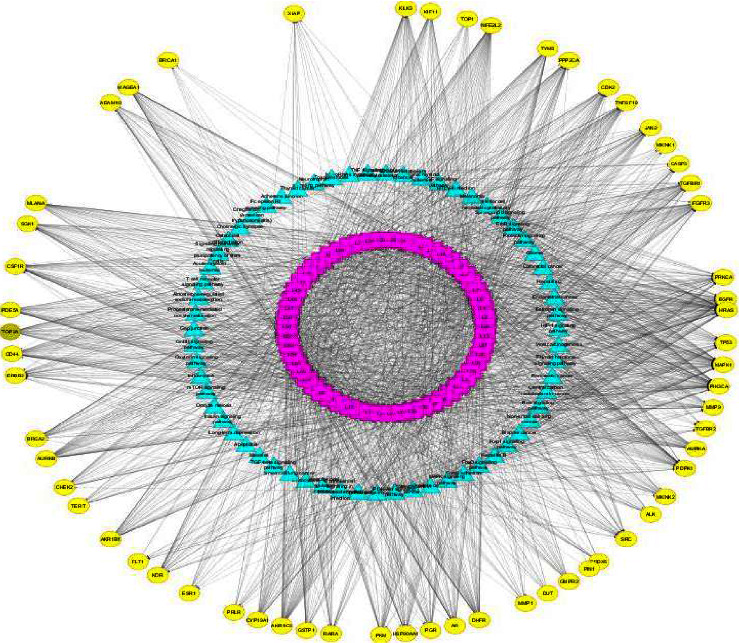
Component–target–pathway network (C-T-P network was constructed by using the “merge” function of cytoscape software).

The C-T-P network was constructed based on active compounds, target identification, and pathway analysis. This network consists of 118 nodes and 1927 edges with nodes corresponding to compounds, targets and pathways, and edges indicating its interactions (Figure 9). This network suggests that compounds L2 (17-hydroxyteuvincenone G), L51 (Scutellarein) and L54 (Teuvincenone A), L10 (Acacetin), L55 (Teuvincenone E), and L50 (Scutellarein 7-glucuronide) possess potential pharmacological activities against cancer and could further play an important role in cancer treatment.

According to the results of compound-target-protein network analysis, proteins with high degree including AKR1C3, DHFR, NFE2L2, AURKB, CD44, PIK3CA, FGFR3, CSF1R, and HRAS were identified as key targets of *Clerodendrum* in the treatment of cancer that which could intervene the potential pathway such as pathway in cancer, PI3K-Akt signaling pathway, proteoglycans in cancer, MAPK signalling pathway, focal adhesion, and prostate cancer for treating cancer in future.

### 3.6. MD Simulation of Scutellarein and 17-Hydroxyteuvincenone*G* with 1ry0 Cancer Target

To study the stability of protein-ligand complexes, MD simulations were performed for 10 ns with the top 2 NCs (Scutellarein and 17-hydroxyteuvincenone G) and 1ry0 cancer target along with PG2 the co-crystallized ligand as the standard. The MD trajectories of Scutellarein-1ry0 and 17-hydroxyteuvincenoneG-1ry0 complexes were compared with co-crystallize ligand prostaglandin D2 (PG2)-1ry0 and values of RMSD, RMSF, Rg, and H-bond were recorded. The complex with a lesser RMSD value could be more stable as compared to higher RMSD values.

**Figure 10:**
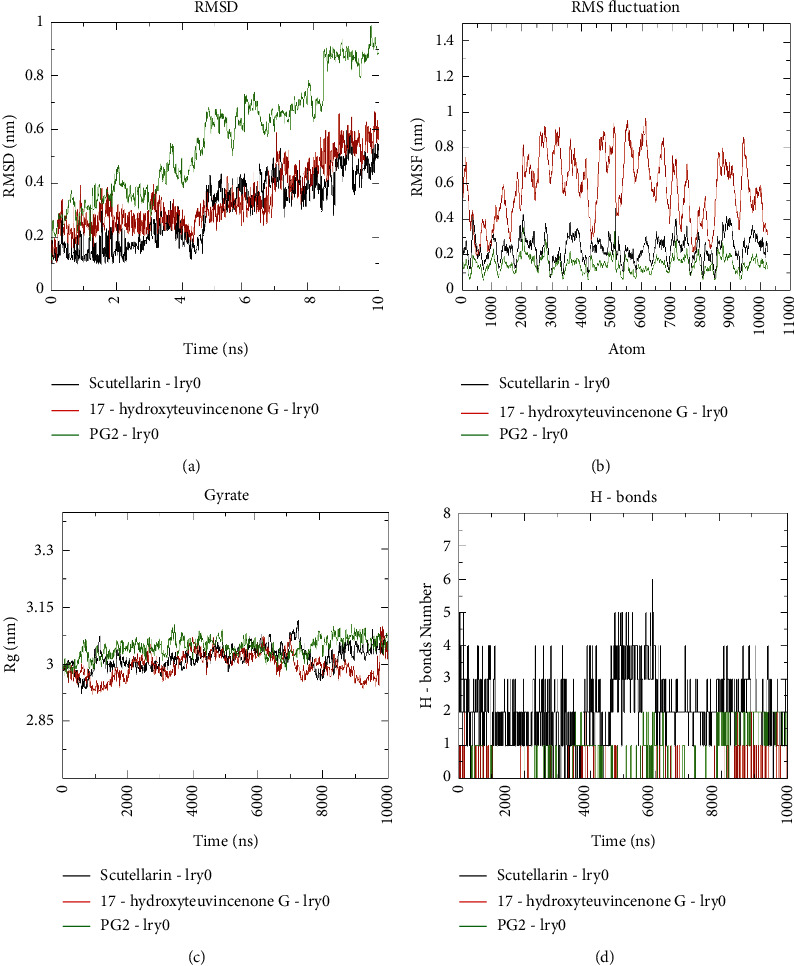
(a) RMSD of backbone atoms for 1ry0-ligands complexes, (b) RMSF of backbone atoms of 1ry0-ligands complexes, (c) Radius of gyration (Rg) of backbone atoms of 1ry0-ligands complexes and (d) Estimation of hydrogen bonds number during the 10 ns over 10 ns simulation of 1ry0-ligands complexes.

The average RMSD values for Scutellarein-1ry0 and 17-hydroxyteuvincenoneG-1ry0 complexes were found to be 0.29 nm and 0.34 nm, respectively, which are significantly stable as compared to the native co-crystal ligand PG2 (0.56 nm). The Scutellarein-1ry0and17-hydroxyteuvincenoneG-1ry0 complexes had a lower RMSD value that range from 0.06 nm to 0.59 nm, 0.07 nm to 0.76 nm, while PG2-1ry0 complexes range from 0.06 nm to 0.98 nm (Figure 10(a)). It indicates that Scutellarein and17-hydroxyteuvincenone*G* formed stable complexes with 1ry0 as compared to co-crystallize ligand PG2. The backbone RMSF of each residue was calculated to access the amino acid residues contributing to the complex structural fluctuations. The average RMSF values of Scutellarein-1ry0, 17-hydroxyteuvincenoneG-1ry0 and PG2-1ry0were 0.14 nm, 0.23 nm, and 0.57 nm, respectively. From the graph Figure 10(b), we could notice that there was an overall decrease in the fluctuation of Scutellarein-1ry0 as compared to 17-hydroxyteuvincenone*G* and PG2. This suggests that Scutellarein was more stable and rigid than the 17-hydroxyteuvincenone*G* structure and PG2.

The analysis of Rg is considered as root mean square distances from each atom of the system to its centre of mass. As depicted in Figure 10(c), the Rg of Scutellarein had a lower value as compared to 17-hydroxyteuvincenone*G* and PG2, which indicates that Scutellarein had a compact and stable complex with 1ry0 during the MD simulation period. Additionally, the H-bonds indicate a crucial role in molecular recognition and determine the stability of the drug-protein complex structure. In Figure 10(d), the intermolecular H-bond trajectories with time-dependent bond distances variation for 10 ns were illustrated. The average number of hydrogen bonds in the standard ligand PG2 (0.13 nm) was less than 17-hydroxyteuvincenoneG-1ry0 (0.58 nm) and Scutellarein-1ry0 (2.12 nm) throughout the MD simulation time period. The lesser number of H-bond in the PG21-1ry0 complex makes it relatively unstable and the higher number of H-bonds in Scutellarein-1ry0 and 17-hydroxyteuvincenoneG-1ry0 represent the stability of the complexes. Overall, the analysis indicates that Scutellarein as compared to 17-hydroxyteuvincenone*G* and PG2 forms a stable protein-ligand complex with 1ry0 cancer target protein and does not depict any conformational change in protein structure during the simulation process.

## 4. Discussion

Some of the developed cancer therapies have resulted in drug resistance against the synthetic drugs [35]. Therefore, natural products are considered as a potential new source of targets for drug discovery as it had higher structural diversity and could reduce the cost of new drug development due to their availability in nature. Therefore, in this study 194 NCs of *Clerodendrum* sp. were evaluated against 60 reported human cancer proteins. All the compounds were initially screened based on molecular descriptors and PCA analysis was evaluated to determine the relationship between statistically meaningful conformations sampled during the trajectory. ADMET profiling of compounds was considered as significant features for drug development [36]. The parameters such as Blood Brain Barrier Penetration (BBB), Caco2 cell permeability, Human Intestinal Absorption (HIA), Madin-Darby canine kidney cells (MDCK), Plasma Protein Binding (PPB), and Skin Permeability (SP) were selected to screen ADME properties of compounds. For toxicity prediction, parameters such as Mutagenic, Tumorigenic, Irritant, Reproductive effective, Druglikness, and Drug score were used. A total of 58 NCs were found to be ADME/toxicity positive drug-like compounds for further study.

The possible mechanism of action and signaling pathways associated with the 60 cancer genes were elucidated with enrichment analysis. The GO enrichment analysis of cancer proteins recorded 29 charts, 45 charts, and 161 charts for CC, MF, and BP analysis. KEGG pathway analysis was performed to examine the signaling pathways and functions of the identified target genes [37]. The analysis of KEGG pathways found that 63 pathways were significantly correlated with target genes. The highest enrichment scores in cancer pathways were obtained in the PI3K-Akt signaling pathway, proteoglycan in cancer, and prostate cancer. The pathway in cancer depicts similar significance in cancer apoptosis, metastasis, cell proliferation, survival, and angiogenesis [38]. The activation of PI3K triggers the phosphorylation of PIP2 to PIP3 that phosphorylates AKT to regulate the metabolism of insulin [39]. The PI3K-Akt signaling pathway involves tumor cell apoptosis and autophagy [40]. These results supported the application of *Clerodendrum* compounds in treating cancer. PPI networks were constructed with 52 nodes and 149 edges and indicated 22 top hub genes with the highest degree in MAPK1, TP53, HSP90AA1, SRC, PIK3CA, HRAS, EGFR, ESR1, ERBB2, PRKCA, AURKA, BRCA1, AR, PGR, JAK2, PDPK1, PPP2CA, TYMS, TOP2A, AURKB, CDK2, and TERT.

**Figure 11:**
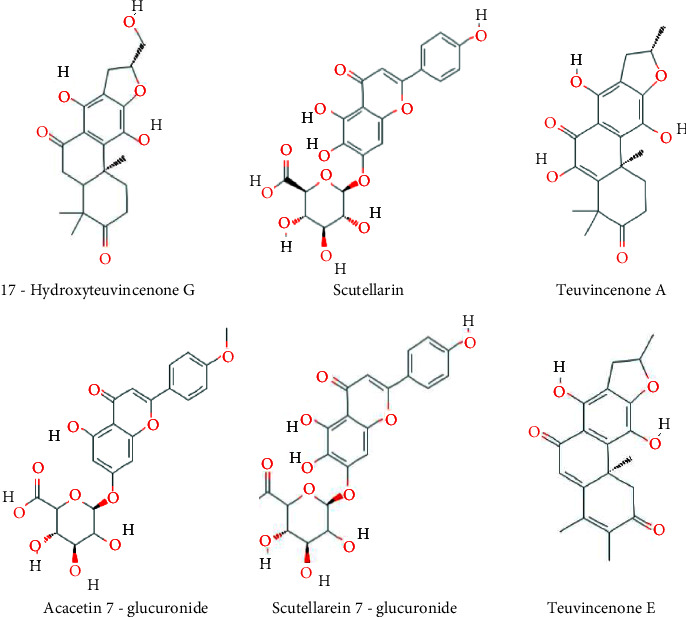
Structure of compounds with potential pharmacological activities against cancer.

**Figure 12:**
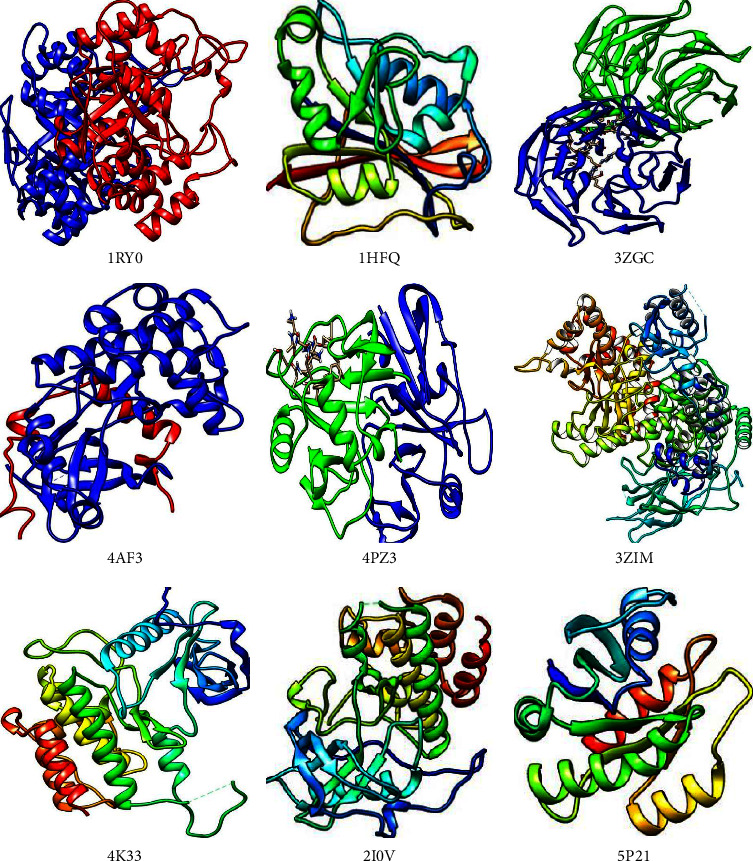
Proteins with highest degree could be used as key target of *Clerodendrum* sp. In the treatment of cancer.

The docking weighted network pharmacology approach was employed to explore the polypharmacological effects of *Clerodendrum* sp. compounds in the treatment of cancer by analysing network interaction between C-T, T-P, and C-T-P. The network between the C-T was evaluated using degree and betweenness centrality, which indicates the number of related nodes in the network. The C-T network suggested that 6 compounds L51 (Scutellarein), L2 (17-hydroxyteuvincenone G), L54 (Teuvincenone A), L10 (Acacetin), L55 (Teuvincenone E), and L50 (Scutellarein 7-glucuronide) had potential pharmacological activities against cancer (Figure 11). This study showed similar comparisons with various works of literature in citing these compounds as anticancer agents as such the compound Scutellarein and Scutellarein 7-glucuronide, flavones glycoside was reported to exhibit antiproliferative and antiapoptotic activities among various human malignancies [41, 42]. Similarly, 17-hydroxyteuvincenone*G* showed significant cytotoxicities activities against the growth of human promyelocyticleukemia (HL-60) and human lung adenocarcinoma epithelial (A-549) tumor cell lines [43]. Teuvincenone A and *E* had remarkable in-vitro cytotoxicity activity against human cell lines [44], and acacetin exerts an antiproliferative effect by blocking cell cycle progression and could enhance the therapeutic potential in nonsmall cell lung cancer cell [45] and breast cancer [46]. The nine target proteins with high degree including AKR1C3 (Aldo-ketoreductase family 1 member C3), DHFR (Dihydrofolate Reductase), NFE2L2 (Nuclear factor erythroid 2-related factor 2-Like 2), AURKB (Aurora B Kinase), CD44 (CD44 antigen), PIK3CA (Phosphatidylinositol 4,5-bisphosphate 3-kinase catalytic subunit alpha isoform), FGFR3 (Fibroblast growth factor receptor 3), CSF1R (Macrophage colony-stimulating factor 1 receptor), and HRAS (GTPaseHras) were identified as a key target of *Clerodendrum* sp. in the treatment of cancer (Figure 12). Therefore, targeting these mutant proteins with novel therapeutics agents could eliminate the morbidity and mortality of human cancer.

The network analysis of T-P and C-T-P supports different pathways such as Pathway in cancer, PI3K-Akt signaling pathway, Proteoglycans in cancer, MAPK signaling pathway, Focal adhesion, and Prostate cancer as potential signaling pathways to mediate the significant effects of *Clerodendrum* compounds against cancer.

MD simulation could determine the underlying dynamics of protein-ligand interactions [47]. Based on Network pharmacology, the MD simulation was performed for 10 ns with PG2 co-crystallize ligand as standard and determined the stability of NCs with the cancer target protein. The top 2 bioactive compounds (Scutellarein and 17-hydroxyteuvincenone G) had maximum interactions with cancer targets and the 1ry0 cancer target based on the highest docking scores was used as starting point for MD simulation analysis.The analysis of MD trajectories revealed that Scutellarein and17-hydroxyteuvincenone*G* had favourable conformational stability, flexibility, and binding energy when docked with 1ry0 as compared to the co-crystallized structure of the standard 1ry0-PG2 complex. Therefore, Scutellarein and17-hydroxyteuvincenone*G* could be proposed as effective compounds in inhibition of protein target for further in-vitro and in-vivo anticancer studies. Many studies suggest that modulation of multi-targets rather than single targets could lead to discovering effective drugs [48]. Hence, these findings could benefit from the current knowledge of drug-protein interaction and relate pharmacological space with genomic space in order to treat cancer disease.

## 5. Conclusions

A total of 58 compounds were screened based on molecular descriptors, PCA analysis, and ADME/toxicity. The 60 cancer target genes were analysed with GO annotation, KEGG pathway, and PPI interactions. The selected NCs and cancer targets were analysed with network-related tools to confirmed and reveal the potential anti-cancer activity and molecular mechanism of *Clerodendrum* compounds. The result of Network pharmacology analysis revealed that 6 active compounds exert their anti-cancer activity with 9 targets in 63 pathways. These results suggested that the identified compounds might contribute to regulate with different cancer related genes and signaling pathways with potential applications in cancer treatment. Further in-vivo experimental studies are still in demands to validate our findings as this study was performed based on data analysis and contributes to scavenger new source of compounds and the development of new anti-cancer drugs.

## Figures and Tables

**Table 1:** Statistical parameters of 8 molecular descriptors in NCs and Drugs Library.

Descriptor Variable	NPs (194 molecules)					Drugs (46 molecules)				
	Min	Max	Mean	Median	Std. Dev.	Min	Max	Mean	Median	Std. Dev.
ALogP	−8.80	3.58	−1.33	−1.12	2.33	−3.38	11.57	−0.70	−1.17	2.52
Molecular weight	110.04	1062.50	398.10	387.20	172.82	150.10	853.33	414.53	361.17	191.21
Num H-Donors	0.00	13.00	3.05	2.00	2.74	0.00	15.00	4.76	3.00	4.28
Num H-Acceptors	0.00	23.00	4.98	3.00	4.89	0.00	6.00	2.34	2.00	1.75
Num rotatable bonds	0.00	33.00	5.86	4.00	6.03	0.00	17.00	5.33	4.00	4.55
Num of rings	0.00	10.00	3.28	2.00	1.71	0.00	9.00	3.78	3.00	2.32
Lipinski failure	0.00	3.00	0.84	1.00	0.96	0.00	3.00	0.54	0.00	0.82
Total polar surface area	0.00	327.6	104.28	84.40	75.93	0.00	224.45	97.50	89.06	54.68

**Table 2:** ADME/Toxicity profiling of 58 NCs using PreADMET server and Osiris Property Explorer.

Sl no.	Name	ADME properties						Toxicity poperties					
		BBB	Caco_2_	HIA	MDCK	PPB	SP	Mutagenic	Tumorigenic	Irritant	Reproductive effective	Drug-likeness	Drug-score
L1	17-Hydroxyteuvincen-5(6)-enone G	0.08	21.06	87.65	112.07	78.43	−3.39	Green	Green	Green	Green	−5.39	0.4
L2	17-Hydroxyteuvincenone G	0.08	21.06	86.22	82.68	83.04	−4.48	Green	Green	Green	Green	−6.39	0.39
L3	3-4-Dihydroxyphenylethanol	0.87	19.36	79.62	93.87	3.04	−3.28	Green	Green	Green	Green	−1.3	0.59
L4	3′-hydroxy-3,4-dimethoxychalcone	0.16	37.44	95.58	26.08	90.00	−2.45	Green	Green	Green	Green	3.18	0.81
L5	5-Hydroxy-3′,4′,7-trimethoxyflavone	0.01	31.47	96.48	1.94	87.11	−3.44	Green	Green	Green	Green	3.26	0.8
L6	6,7-Dehydroroyleanone	1.37	22.52	95.46	224.09	100	−2.23	Green	Green	Green	Green	−7.61	0.39
L7	7-Hyroxy flavanone	0.81	13.49	95.23	39.39	94.35	−3.04	Green	Green	Green	Green	0.37	0.68
L8	Acacetin7-glucuronide	0.15	12.79	93.04	20.23	90.91	−3.36	Green	Green	Green	Green	−1.47	0.51
L9	Acacetin 7-glucoside	0.03	7.73	65.89	0.16	68.77	−4.51	Green	Green	Green	Green	−4.87	0.39
L10	Acacetin	0.01	8.50	50.56	0.075	74.30	−4.36	Green	Green	Green	Green	−1.35	0.46
L11	Apigenin 7,4′-dimethyl ether	0.08	34.57	95.99	8.56	88.96	−3.30	Green	Green	Green	Green	−1.47	0.49
L12	Apigenin-7-O-glucoside	0.03	7.21	47.10	0.64	73.43	−4.52	Green	Green	Green	Green	−2.29	0.44
L13	Arabinose	0.11	0.56	32.86	0.60	1.49	−4.63	Green	Green	Green	Green	−2.87	0.52
L14	Astragalin	0.035	11.14	25.17	1.14	57.57	−4.64	Green	Green	Green	Green	−2.68	0.42
L15	Aucubin	0.05	9.93	20.68	0.53	25.22	−5.06	Green	Green	Green	Green	−4.15	0.47
L16	Aurantiamide	1.60	21.66	93.43	67.00	95.74	−2.75	Green	Green	Green	Green	1.74	0.64
L17	Baicalein	0.77	1.28	88.10	101.90	98.98	−4.13	Green	Green	Green	Green	0.75	0.75
L18	Cabreuvin	0.01	45.21	97.92	2.71	85.23	−3.38	Green	Green	Green	Green	1.69	0.76
L19	Calceolarioside A	0.04	13.51	29.56	0.04	78.78	−3.68	Green	Green	Green	Green	−9.86	0.38
L20	Chrysoeriol	0.08	5.18	88.18	37.45	90.87	−4.14	Green	Green	Green	Green	1.46	0.8
L21	Cirsimaritin	0.05	8.36	93.37	7.45	88.05	−3.43	Green	Green	Green	Green	1.11	0.74
L22	Clerodenone A	0.02	12.44	85.91	0.54	38.36	−4.94	Green	Green	Green	Green	1.62	0.86
L23	Clerodermic acid	1.05	20.97	97.93	258.85	96.39	−2.61	Green	Green	Green	Green	−3.42	0.41
L24	Cleroflavone	0.10	39.60	95.99	0.79	89.17	−3.21	Green	Green	Green	Green	−1.69	0.45
L25	Cleroindicin A	0.53	30.26	95.93	7.93	90.43	−3.48	Green	Green	Green	Green	−1.21	0.6
L26	Cleroindicin B	0.27	17.36	81.83	1.00	1.22	−3.74	Green	Green	Green	Green	-4.68	0.49
L27	Cleroindicin C	0.33	11.48	89.78	0.81	14.96	−4.16	Green	Green	Green	Green	−0.31	0.69
L28	Cleroindicin D	0.24	11.99	77.58	0.57	9.07	−5.15	Green	Green	Green	Green	1.42	0.89
L29	Cleroindicin E	0.16	12.17	69.89	0.55	7.32	−5.17	Green	Green	Green	Green	0.64	0.81
L30	Cleroindicin F	0.34	11.48	91.29	0.88	34.92	−4.08	Green	Green	Green	Green	1.62	0.9
L31	Corchorifatty acid E	0.27	12.61	89.70	22.85	100	−1.21	Green	Green	Green	Green	−16.08	0.46
L32	Cynaroside	0.03	4.87	25.16	0.75	73.27	−4.60	Green	Green	Green	Green	−1.79	0.45
L33	Cyrtophyllone B	0.56	20.41	88.37	213.79	97.09	−2.61	Green	Green	Green	Green	−7.59	0.35
L34	Ethyl caffeate	0.19	21.28	89.45	181.26	61.02	−2.14	Green	Green	Green	Green	−6.78	0.48
L35	Formidiol	0.12	21.01	94.56	13.61	90.97	−3.63	Green	Green	Green	Green	−3.45	0.35
L36	Hispidulin	0.10	5.46	88.18	32.22	91.55	−4.12	Green	Green	Green	Green	1.11	0.77
L37	Hispidulin-7-o-glucoronide	0.02	11.19	27.52	0.08	66.72	−4.42	Green	Green	Green	Green	1.18	0.66
L38	Indole-3-carboxylic acid	1.28	20.66	88.51	57.90	30.28	−3.90	Green	Green	Green	Green	1.78	0.88
L39	Isorhamnetin 3-O-glucoside	0.03	9.93	21.60	0.53	47.83	−4.70	Green	Green	Green	Green	−2.37	0.41
L40	Lariciresinol	0.29	22.67	89.81	1.81	84.63	−3.58	Green	Green	Green	Green	0.96	0.74
L41	Leonuriside A	0.07	9.81	32.36	2.46	35.98	−4.72	Green	Green	Green	Green	−3.49	0.48
L42	Luteoline	0.36	4.53	79.42	36.52	99.71	−4.28	Green	Green	Green	Green	1.9	0.84
L43	Methyl caffeate	0.67	21.13	88.69	103.64	50.36	−2.44	Green	Green	Green	Green	−2.77	0.51
L44	Rengyol	0.35	17.42	73.29	1.57	40.00	−3.41	Green	Green	Green	Green	−2.64	0.62
L45	Rosesoide	0.04	10.89	46.82	0.07	41.86	−4.15	Green	Green	Green	Green	−3.82	0.46
L46	Salidroside	0.05	5.38	48.08	0.86	41.75	−4.53	Green	Green	Green	Green	−3.79	0.48
L47	Sammangaoside A	0.04	8.35	44.66	0.04	37.18	−5.02	Green	Green	Green	Green	−2.8	0.46
L48	Sammangaoside B	0.04	8.34	39.80	0.04	40.67	−5.25	Green	Green	Green	Green	−4.26	0.44
L49	Scutellarein 4′-methyl ether	0.16	7.01	88.17	6.37	92.80	−4.17	Green	Green	Green	Green	−1.68	0.51
L50	Scutellarein 7-glucuronide	0.02	10.13	15.78	0.10	74.98	−4.46	Green	Green	Green	Green	1.04	0.67
L51	Scutellarin	0.02	10.13	15.78	0.10	74.98	−4.46	Green	Green	Green	Green	1.04	0.67
L52	Sorbifolin	0.17	7.01	88.18	13.17	91.49	−4.16	Green	Green	Green	Green	1.11	0.77
L53	Spicatolignan B	0.03	20.44	92.81	49.11	83.72	−3.23	Green	Green	Green	Green	2.84	0.8
L54	Teuvincenone A	0.07	21.09	87.42	143.85	81.06	−4.14	Green	Green	Green	Green	−1.68	0.44
L55	Teuvincenone E	0.76	17.37	94.17	206.59	88.61	−3.69	Green	Green	Green	Green	−3.06	0.36
L56	Teuvincenone H	0.22	21.09	89.98	146.03	84.56	−4.08	Green	Green	Green	Green	−4.76	0.34
L57	Trichotomone	0.81	21.50	94.24	0.04	90.15	−2.13	Green	Green	Green	Green	−4.76	0.34
L58	Villosin C	0.09	20.50	80.60	252.46	88.76	−4.39	Green	Green	Green	Green	−6.55	0.39

## Data Availability

The data are available within the manuscript and also accessible from the corresponding author upon request.

## Supplementary Materials

Supplementary Materials Table S1: List of compounds from *Clerodendrum* sp. retrieved through literature for this study. Table S2: List of Cancer Target retrieved from literature and selected for analysis.
