# Supplementary material for: Virtual Screening and Network Pharmacology-Based Study to Explore the Pharmacological Mechanism of Clerodendrum Species for Anticancer Treatment
Source: Evid Based Complement Alternat Med. 2022 Nov 2;2022:3106363. doi: 10.1155/2022/3106363 (PMC9646327; doi:10.1155/2022/3106363)
Supplement: Supplementary Materials — Table S1: List of compounds from Clerodendrum sp. retrieved through literature for this study. Table S2: List of Cancer Target retrieved from literature and selected for analysis. [file 3106363.f1.zip › Table S1.docx]

Table S1: List of compounds from *Clerodendrum* sp. retrieved through literatures for this study

| **Sl No.** | **Compounds** | **Plant Source** | **Structures** |
| --- | --- | --- | --- |
| 1 | 2',4'-dihydroxy-3,4-dimethoxychalcone | *C. inerme* | 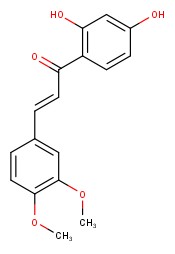 |
| 2 | 3-hydroxy-3,4-dimethoxychalcone | *C. inerme* | 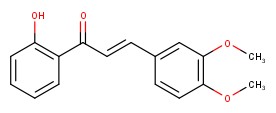 |
| 3 | 5-hydroxy-3',4',7-trimethoxyflavone | *C. inerme* | 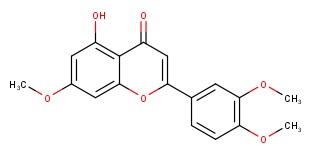 |
| 4 | 7-o-methylwogonin | *C. inerme* | 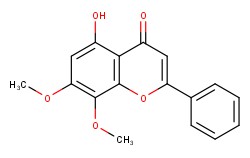 |
| 5 | Acacetin | *C. inerme* | 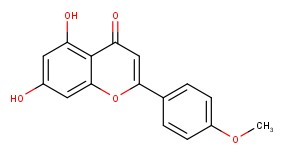 |
| 6 | Acacetin 7-glucoside | *C. inerme* | 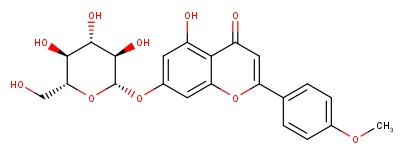 |
| 7 | Acacetin 7-glucuronide | *C. inerme* | 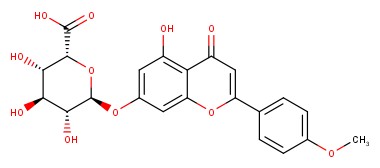 |
| 8 | Apigenin | *C. serratum, C. inerme* | 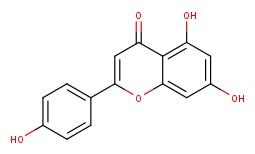 |
| 9 | Apigenin 7,4'-dimethyl ether | *C. inerme* | 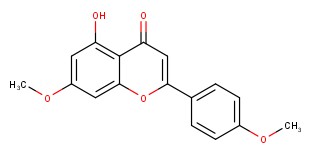 |
| 10 | Apigenin-7-O-glucoside | *C. inerme* | 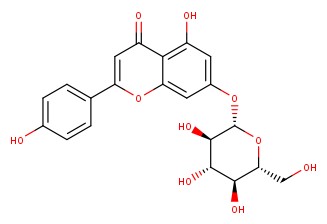 |
| 11 | Astragalin | *C. philippinum* | 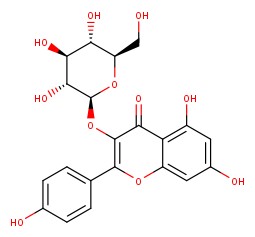 |
| 12 | Baicalein | *C. serratum* | 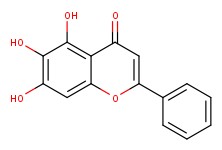 |
| 13 | Cabreuvin | *C. infortunatum* | 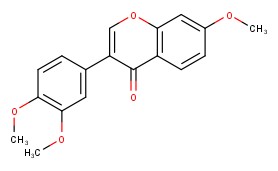 |
| 14 | Catechin | *C. serratum* | 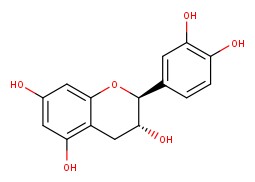 |
| 15 | Chrysoeriol | *C. trichotomum* | 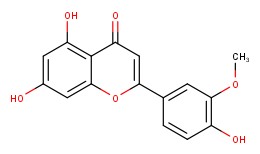 |
| 16 | Cirsimaritin | *C. philippinum* | 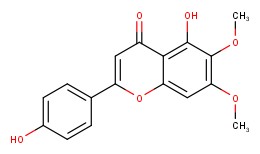 |
| 17 | Clerodendroside A | *C. infortunatum* | 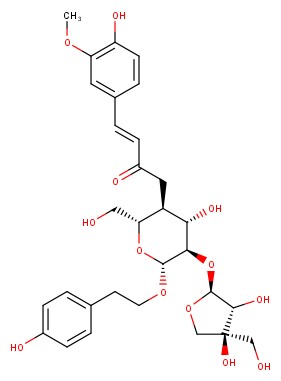 |
| 18 | Cynaroside | *C. inerme* | 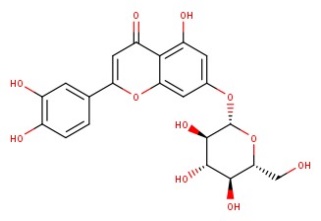 |
| 19 | Eupafolin | *C. indicum* | 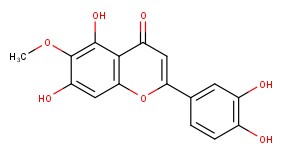 |
| 20 | Eucalyptin | *C. inerme* | 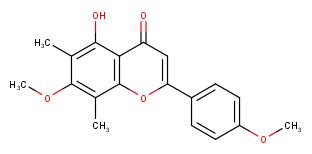 |
| 21 | Flavone | *C. philippinum* | 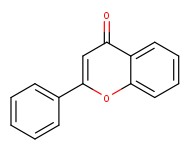 |
| 22 | Hispidulin | *C. indicum* | 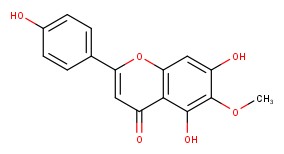 |
| 23 | Hispidulin-7-o-glucuronide | *C. indicum* | 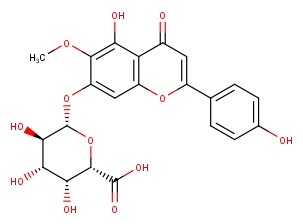 |
| 24 | Isorhamnetin 3-o-glucoside | *C. trichotomum* | 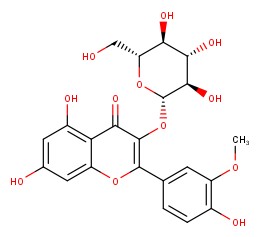 |
| 25 | Kaempferol | *C. fragnans* | 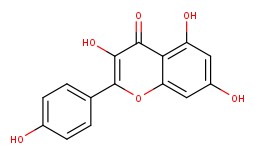 |
| 26 | Luteoline | *C. serratum* | 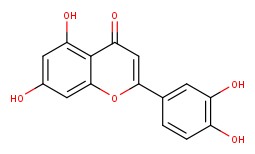 |
| 27 | Markhamioside F | *C. inerme* | 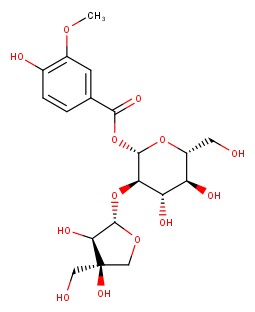 |
| 28 | Pectolinarigenin | *C. indicum* | 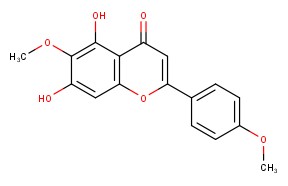 |
| 29 | Purpureaside | *C. inerme* | 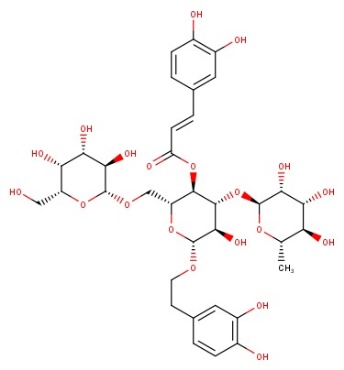 |
| 30 | Quercetin | *C. infortunatum* | 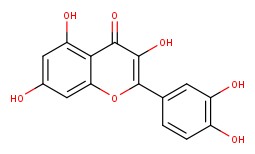 |
| 31 | Salvigenin | *C. inerme* | 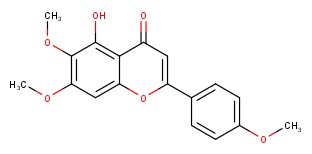 |
| 32 | Sorbifolin | *C. philippinum* | 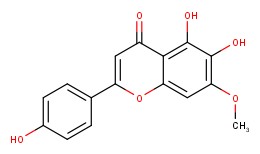 |
| 33 | Tricin | *C. japonicum* | 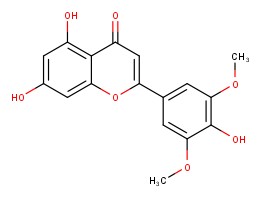 |
| 34 | 7-hyroxyflavanone | *C. serratum* | 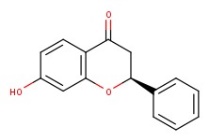 |
| 35 | 5-o-butyl cleroindin D | *C. infortunatum* | 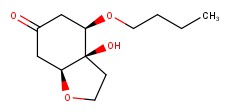 |
| 36 | Cleroindicin A | *C. indicum* | 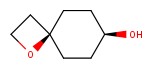 |
| 37 | Cleroindicin B | *C. indicum* | 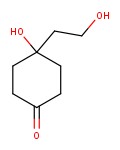 |
| 38 | Cleroindicin C | *C. indicum* | 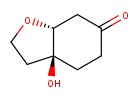 |
| 39 | Cleroindicin D | *C. indicum* | 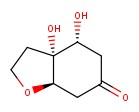 |
| 40 | Cleroindicin E | *C. indicum* | 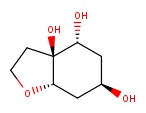 |
| 41 | Cleroindicin F | *C. indicum* | 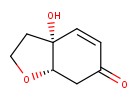 |
| 42 | Uncinatone | *C. indicum* | 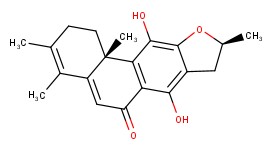 |
| 43 | Genistein | *C. inerme* | 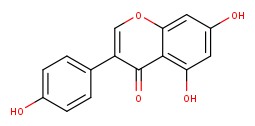 |
| 44 | Genistein 7-O-glucoside | *C. infortunatum* | 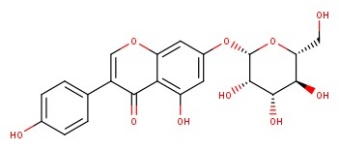 |
| 45 | Scutellarin | *C. infortunatum* | 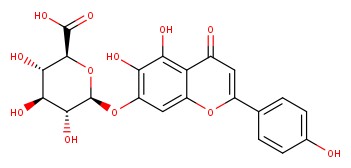 |
| 46 | Scutellarein 4'-methyl ether | *C. inerme* | 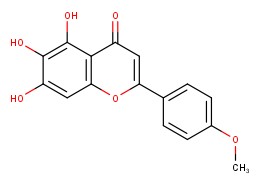 |
| 47 | Scutellarein 7-glucuronide | *C. infortunatum* | 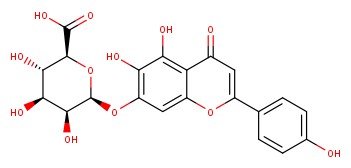 |
| 48 | Scutellarein 7-o-β-d-glucuronate | *C. serratum* | 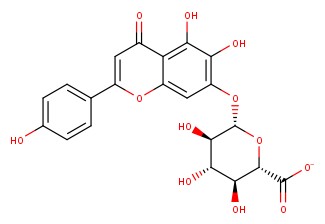 |
| 49 | Acteoside | *C. serratum* | 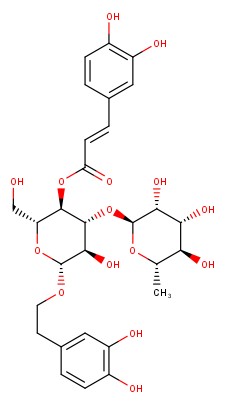 |
| 50 | Caffeic acid | *C. infortunatum* | 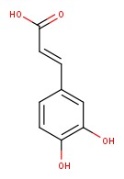 |
| 51 | Campneoside I | *C. inerme* | 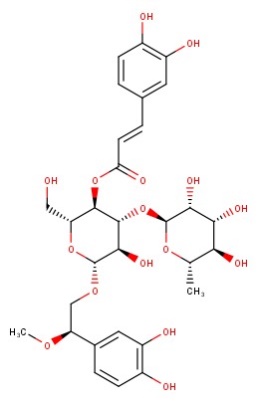 |
| 52 | Calceolarioside A | *C. philippinum* | 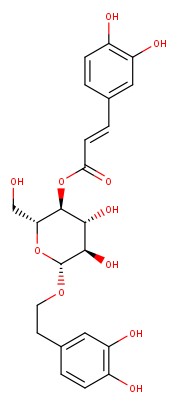 |
| 53 | Cistanoside E | *C. inerme* | 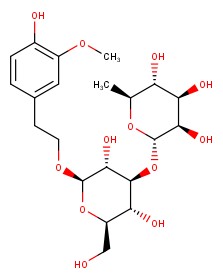 |
| 54 | Clerodenoside A | *C. japonicum* | 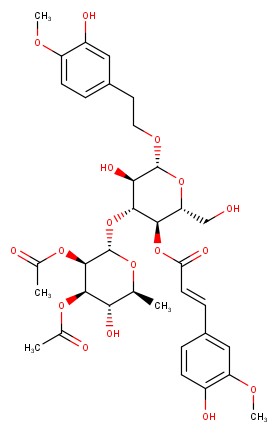 |
| 55 | Darendoside B | *C. inerme* | 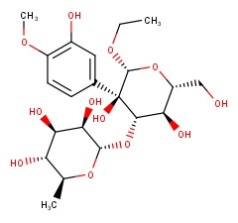 |
| 56 | Ethyl caffeate | *C. philippinum* | 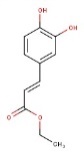 |
| 57 | Ferulic acid | *C. serratum* | 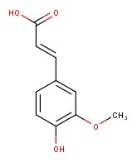 |
| 58 | Isomartynoside | *C. bungei* | 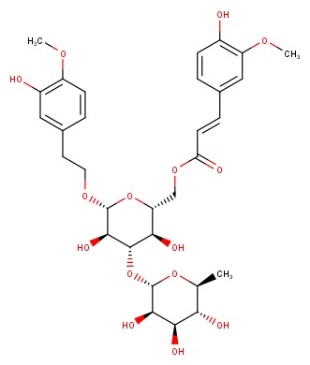 |
| 59 | Isoverbascoside | *C. inerme* | 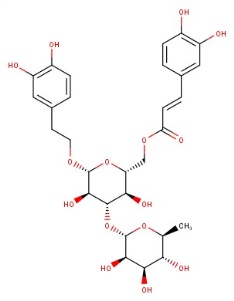 |
| 60 | Jionoside D | *C. trichotomum* | 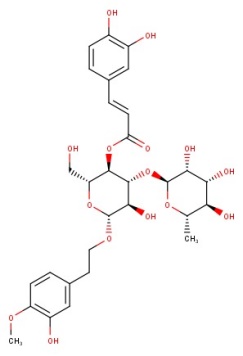 |
| 61 | Leucosceptoside A | *C. bungei* | 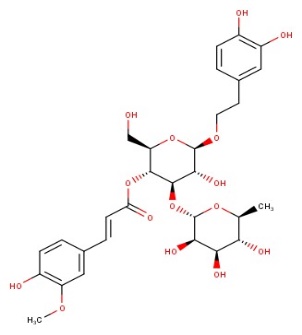 |
| 62 | Martynoside | *C. japonicum* | 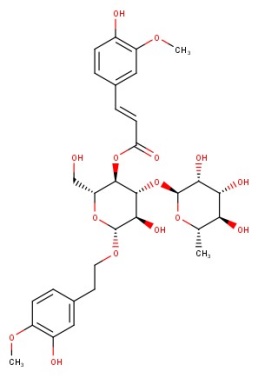 |
| 63 | Methyl caffeate | *C. philippinum* | 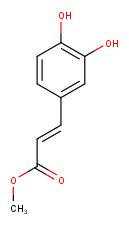 |
| 64 | Trichotomoside | *C. bungei* | 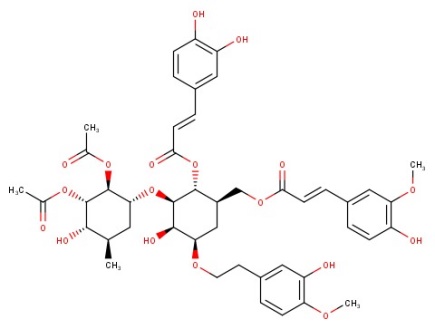 |
| 65 | Dihydrocoumarin | *C. philippinum* | 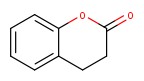 |
| 66 | Osmanthuside | *C. colebrookianum* | 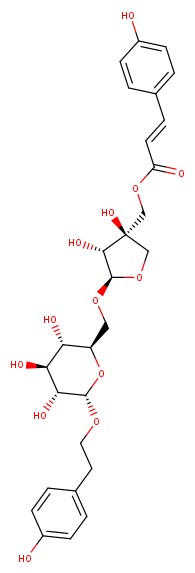 |
| 67 | Dehydrodiconiferyl alcohol 4-O-β-d-glucopyranoside | *C. inerme* | 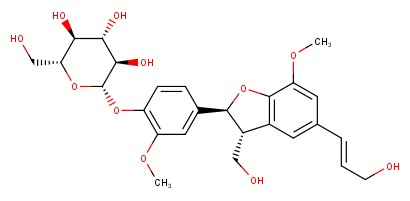 |
| 68 | Dehydrodiconiferyl alcohol 9-O-β-d-glucopyranoside | *C. inerme* | 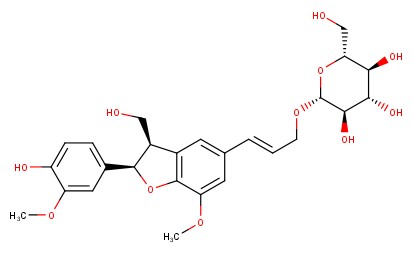 |
| 69 | Lariciresinol | *C. indicum* | 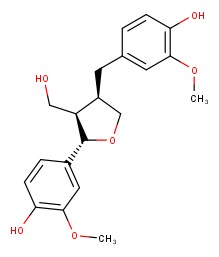 |
| 70 | Lariciresinol-9-O-β-d-glucoside | *C. indicum* | 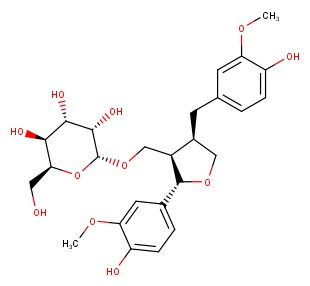 |
| 71 | 8-O-acetylharpagide | *C. thomsoniae* | 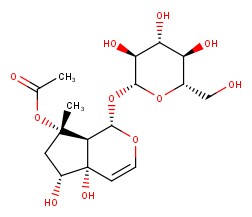 |
| 72 | 8-O-acetylmioporoside | *C. thomsoniae* | 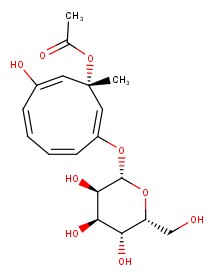 |
| 73 | Aucubin | *C. thomsoniae* | 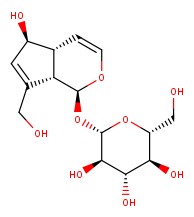 |
| 74 | Harpagide | *C. thomsoniae* | 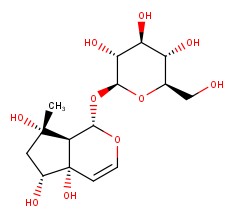 |
| 75 | Inerminoside A | *C. inerme* | 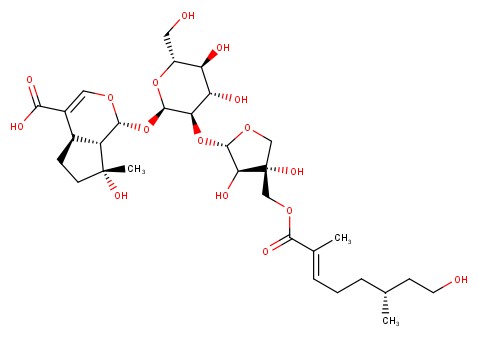 |
| 76 | Inerminoside B | *C. inerme* | 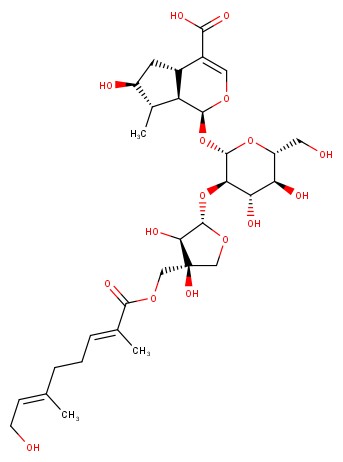 |
| 77 | Inerminoside C | *C. inerme* | 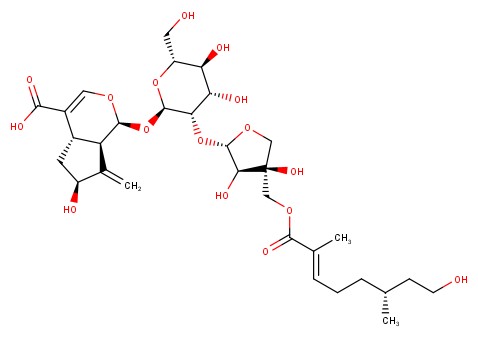 |
| 78 | Inerminoside C heptaacetate | *C. inerme* | 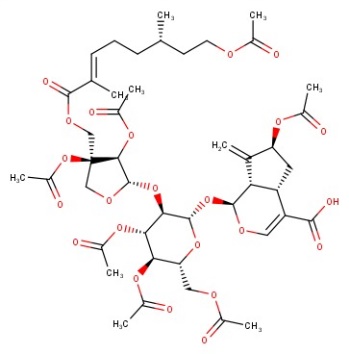 |
| 79 | Inerminoside D | *C. inerme* | 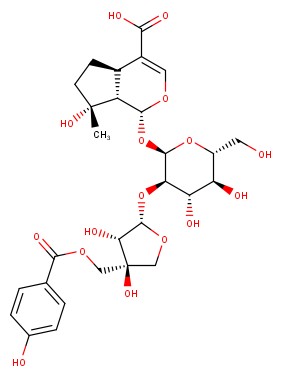 |
| 80 | Inermes A | *C. inerme* | 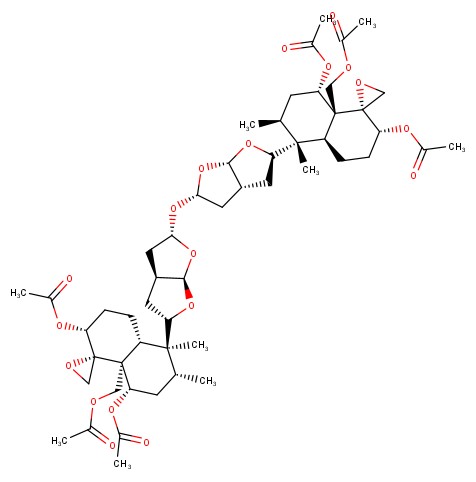 |
| 81 | Inermes B | *C. inerme* | 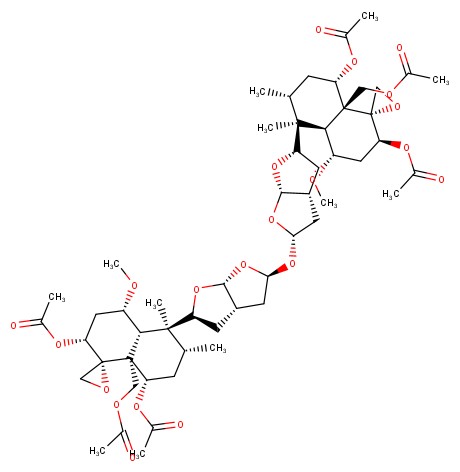 |
| 82 | Linalool | *C. inerme* | 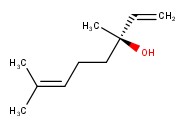 |
| 83 | Megastigmane | *C. inerme* | 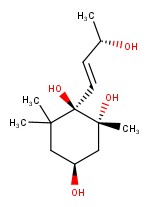 |
| 84 | Monomelittoside | *C. inerme* | 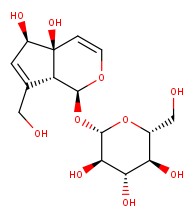 |
| 85 | Reptoside | *C. thomsoniae* | 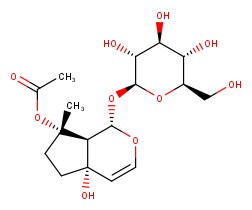 |
| 86 | Sammangaoside A | *C. inerme* | 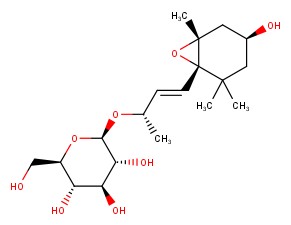 |
| 87 | Sammangaoside B | *C. inerme* | 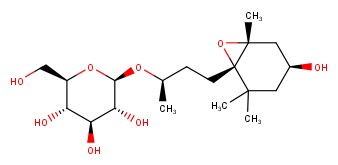 |
| 88 | Sammangaoside C | *C. inerme* | 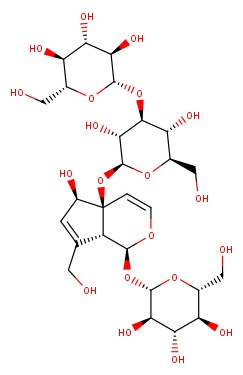 |
| 89 | 14,15-dihydro-15β-methoxy-3-epicaryoptin | *C. inerme* | 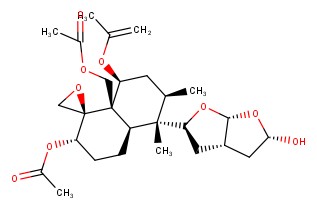 |
| 90 | 6,7-dehydroroyleanone | *C. inerme* | 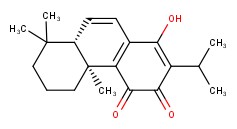 |
| 91 | 14,15-dihydro-15-hydroxy-3-epicaryoptin | *C. inerme* | 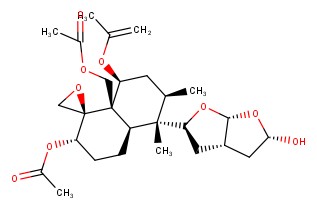 |
| 92 | 17-hydroxyteuvincen-5(6)-enone G | *C. indicum* | 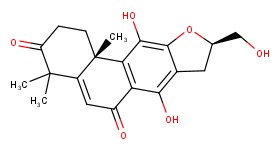 |
| 93 | 17-hydroxyteuvincenone G | *C. indicum* | 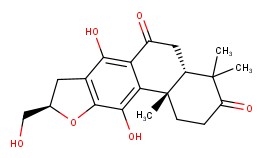 |
| 94 | 18-hydroxy-6-methoxyvillosin C | *C. trichotomum* | 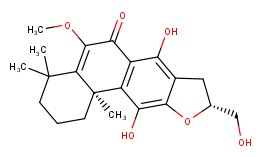 |
| 95 | Clerodin | *C. trichotomum* | 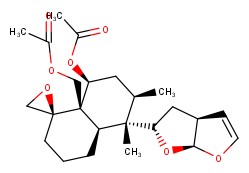 |
| 96 | Clerodermic acid | *C. inerme* | 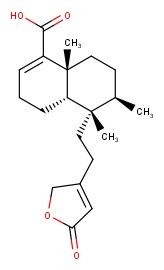 |
| 97 | Clerodendrin A | *C. trichotomum* | 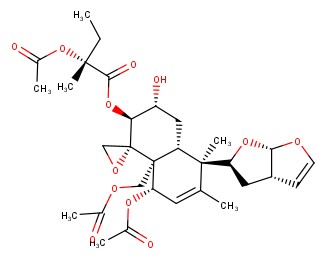 |
| 98 | Clerodendrin B | *C. trichotomum* | 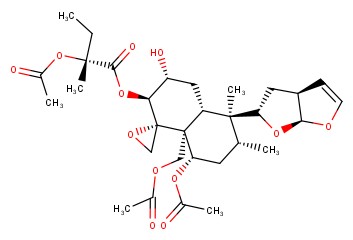 |
| 99 | Clerodendrin C | *C. trichotomum* | 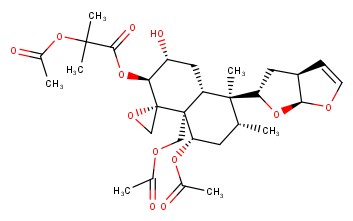 |
| 100 | Clerodendrin D | *C. trichotomum* | 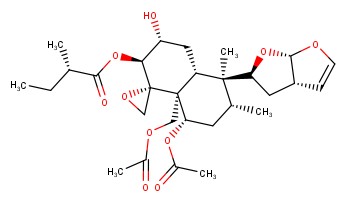 |
| 101 | Clerodendrin E | *C. trichotomum* |  |
| 102 | Clerodendrin F | *C. trichotomum* |  |
| 103 | Clerodendrin G | *C. trichotomum* |  |
| 104 | Clerodendrin H | *C. trichotomum* |  |
| 105 | Cyrtophyllone B | *C. trichotomum* |  |
| 106 | Formidiol | *C. trichotomum* |  |
| 107 | Mandarone E | *C. bungei* |  |
| 108 | Royleanone | *C. inerme* |  |
| 109 | Teuvincenone A | *C. trichotomum* |  |
| 110 | Teuvincenone B | *C. trichotomum* |  |
| 111 | Teuvincenone E | *C. trichotomum* |  |
| 112 | Teuvincenone H | *C. trichotomum* |  |
| 113 | Trichotomone | *C. trichotomum* |  |
| 114 | Sugiol | *C. trichotomum* |  |
| 115 | 3-O-acetyloleanolicacid | *C. inerme* |  |
| 116 | 4α-methyl-24β-ethyl-5α-cholesta-14 | *C. inerme* |  |
| 117 | α-amyrin | *C. philippinum* |  |
| 118 | β-amyrin | *C. inerme* |  |
| 119 | Betulin | *C. infortunatum, C. inerme, C. trichotomum* |  |
| 120 | Betulinic acid | *C. inerme* |  |
| 121 | Clerodenone A | *C. philippinum* |  |
| 122 | Clerodolone | *C. infortunatum* |  |
| 123 | Clerodone | *C. infortunatum* |  |
| 124 | Friedelin | *C. trichotomum* |  |
| 125 | Glutinol | *C. inerme* |  |
| 126 | Glutinone | *C. inerme* |  |
| 127 | Icosahydropicenic acid | *C. serratum* |  |
| 128 | Lupeol | *C. indicum, C. trichotomum, C. inerme* |  |
| 129 | Magnificol | *C. inerme* |  |
| 130 | Maslinic acid | *C. colebrookianum* |  |
| 131 | Obtusifoliol | *C. inerme* |  |
| 132 | Oleanolic acid | *C. serratum* |  |
| 133 | Queretaroic acid | *C. serratum* |  |
| 134 | Serratagenic acid | *C. serratum* |  |
| 135 | Rosesoide | *C. indicum* |  |
| 136 | Taraxerol | *C. indicum* |  |
| 137 | Melittoside | *C. trichotomum* |  |
| 138 | 9-hydroxytridecyl docosanoate | *C. philippinum* |  |
| 139 | Lignoceric acid | *C. infortunatum* |  |
| 140 | Oleic acid | *C. infortunatum* |  |
| 141 | Palmitic acid | *C. trichotomum* |  |
| 142 | Stearic acid | *C. infortunatum* |  |
| 143 | Tetracosanoic acid | *C. infortunatum* |  |
| 144 | 11-pentacosanone | *C. inerme* |  |
| 145 | N decanol | *C. trichotomum* |  |
| 146 | Corchorifatty acid E | *C. trichotomum* |  |
| 147 | Lenoleic | *C. infortunatum* |  |
| 148 | 22-dehydroclerosterol | *C. trichotomum* |  |
| 149 | α-spinosterol | *C. serratum* |  |
| 150 | β-sitosterol | *C. trichotomum, C. inerme* |  |
| 151 | Clerosterol | *C. indicum* |  |
| 152 | Gamma-sitosterol | *C. trichotomum* |  |
| 153 | Stigmasterol | *C. indicum, C. serratum* |  |
| 154 | 24-methylenelophenol | *C. inerme* |  |
| 155 | (24s)-24-methyl-25-dehydrocholesterol | *C. inerme* |  |
| 156 | Campesterol | *C. serratum* |  |
| 157 | 24-ethyl cholesterol | *C. serratum* |  |
| 158 | 24-methylcholestanol | *C. inerme* |  |
| 159 | 24-methyl-lathosterol | *C. inerme* |  |
| 160 | Cholestanol | *C. inerme* |  |
| 161 | Sterol | *C. infortunatum* |  |
| 162 | Colebrin A | *C. colebrookianum* |  |
| 163 | Colebrin B | *C. colebrookianum* |  |
| 164 | Colebrin C | *C. colebrookianum* |  |
| 165 | Colebrin D | *C. colebrookianum* |  |
| 166 | Colebrin e | *C. colebrookianum* |  |
| 167 | Monopalmitin | *C. trichotomum* |  |
| 168 | Benzoic acid | *C. colebrookianum* |  |
| 169 | Benzyl acetate | *C. inerme* |  |
| 170 | Benzyl benzoate | *C. inerme* |  |
| 171 | Bis(2-ethylhexyl) phthalate | *C. inerme* |  |
| 172 | Syringic acid | *C. inerme* |  |
| 173 | Hydroquinone | *C. indicum* |  |
| 174 | Phenyl propanol | *C. philippinum* |  |
| 175 | 3-4-dihydroxyphenylethanol | *C. indicum* |  |
| 176 | Aloe-emodin | *C. trichotomum* |  |
| 177 | Anthraquinone | *C. inerme* |  |
| 178 | Chrysophanol | *C. trichotomum* |  |
| 179 | Emodin | *C. trichotomum* |  |
| 180 | 5-hydroxymethylfurfural | *C. infortunatum* |  |
| 181 | Arabinose | *C. serratum* |  |
| 182 | Leonuriside A | *C. trichotomum* |  |
| 183 | Raffinose | *C. infortunatum* |  |
| 184 | Seguinoside K | *C. inerme* |  |
| 185 | Aurantiamide | *C. trichotomum* |  |
| 186 | Fumaric acid | *C. infortunatum* |  |
| 187 | Indole-3-carboxylic acid | *C. trichotomum* |  |
| 188 | Villosin C | *C. bungei* |  |
| 189 | Rengyol | *C. trichotomum* |  |
| 190 | Salidroside | *C. inerme* |  |
| 191 | 1-hydroxy-1-(8-palmitoyloxyethyl) cyclohexanone | *C. trichotomum* |  |
| 192 | 2-methyleicosa 2,9-diene | *C. colebrookianum* |  |
| 193 | 2,9-diene,10,11,32-trimethyltetratriacontanol | *C. colebrookianum* |  |
| 194 | Spicatolignan B | *C. trichotomum* |  |
