# Supplementary material for: Virtual Screening and Network Pharmacology-Based Study to Explore the Pharmacological Mechanism of Clerodendrum Species for Anticancer Treatment
Source: Evid Based Complement Alternat Med. 2022 Nov 2;2022:3106363. doi: 10.1155/2022/3106363 (PMC9646327; doi:10.1155/2022/3106363)
Supplement: Supplementary Materials — Table S1: List of compounds from Clerodendrum sp. retrieved through literature for this study. Table S2: List of Cancer Target retrieved from literature and selected for analysis. [file 3106363.f1.zip › Table S2.docx]

Table S2: List of Cancer Target retrieved from literature and selected for analysis

| Sl no. | Target | | Gene | PDB ID | | Experimental methods | | Resolution(Å) | | Pubmed ID | | Disease |
| --- | --- | --- | --- | --- | --- | --- | --- | --- | --- | --- | --- | --- |
| 1 | 3-phosphoinositide-dependent protein kinase 1 | PDPK1 | | 2R7B | X-Ray Diffraction | | 2.7 | | 17941624 | | Solid tumour/cancer | |
| 2 | Aldo-keto reductase family 1 member C3 | AKR1C3 | | 1RY0 | X-Ray Diffraction | | 1.7 | | 14979715 | | Prostate cancer | |
| 3 | Aldose reductase | AKR1B1 | | 2FZ8 | X-Ray Diffraction | | 1.5 | | 16952371 | | Stomach cancer | |
| 4 | ALK tyrosine kinase receptor | ALK | | 2XBA | X-Ray Diffraction | | 1.9 | | 20695522 | | Non-small-cell lung cancer, Solid tumour/cancer | |
| 5 | Androgen Receptor | AR | | 1GS4 | X-Ray Diffraction | | 1.9 | | 11906285 | | Prostate cancer | |
| 6 | Aromatase | CYP19A1 | | 3S7S | X-Ray Diffraction | | 3.2 | | 22951074 | | Bladder cancer | |
| 7 | Aurora-A Protein Kinase | AURKA | | 1MQ4 | X-Ray Diffraction | | 1.9 | | 12467573 | | Thyroid cancer | |
| 8 | Aurora B Kinase | AURKB | | 4AF3 | X-Ray Diffraction | | 2.7 | | 22920039 | | Fibrolamellar liver cancer | |
| 9 | Breast cancer type 1 susceptibility protein | BRCA1 | | 1T15 | X-Ray Diffraction | | 1.9 | | 15133502 | | Breast cancer | |
| 10 | Breast cancer type 2 susceptibility protein | BRCA2 | | 3EU7 | X-Ray Diffraction | | 2.2 | | 19609323 | | Breast cancer | |
| 11 | Caspase-3 | CASP3 | | 1NME | X-Ray Diffraction | | 1.6 | | 12563278 | | Solid tumour/cancer | |
| 12 | CD44 antigen | CD44 | | 4PZ3 | X-Ray Diffraction | | 1.2 | | 25195884 | | Solid tumour/cancer,Macular degeneration | |
| 13 | Cellular tumor antigen p53 | TP53 | | 2X0V | X-Ray Diffraction | | 1.8 | | 20142040 | | Oral cancer, Solid tumour/cancer | |
| 14 | cGMP-specific 3',5'-cyclic phosphodiesterase | PDE5A | | 1RKP | X-Ray Diffraction | | 2.1 | | 14668322 | | Colorectal cancer | |
| 15 | Cyclin-dependent kinase 2 | CDK2 | | 1DI8 | X-Ray Diffraction | | 2.2 | | 10633045 | | Non-small-cell lung cancer | |
| 16 | Deoxyuridine 5'-triphosphate nucleotidohydrolase, mitochondrial | DUT | | 1Q5H | X-Ray Diffraction | | 2.0 | | 8805593 | | Solid tumour/cancer; Non-small-cell lung cancer | |
| 17 | Dihydrofolate Reductase | DHFR | | 1HFQ | X-Ray Diffraction | | 2.1 | | 9627670 | | Solid tumour/cancer | |
| 18 | Disintegrin and metalloproteinase domain-containing protein 10 | ADAM10 | | 6BE6 | X-Ray Diffraction | | 2.8 | | 29224781 | | Solid tumour/cancer; Breast cancer | |
| 19 | DNA topoisomerase 1 | TOP1 | | 1A35 | X-Ray Diffraction | | 2.5 | | 9488644 | | Solid tumour/cancer; Breast cancer | |
| 20 | DNA topoisomerase 2-alpha | TOP2A | | 1ZXM | X-Ray Diffraction | | 1.9 | | 16100112 | | Solid tumour/cancer | |
| 21 | E3 ubiquitin-protein ligase XIAP | XIAP | | 4KJU | X-Ray Diffraction | | 1.6 | | 24093940 | | Haematological malignancy, Solid tumour/cancer | |
| 22 | Epidermal Growth Factor Receptor | EGFR | | 1M17 | X-Ray Diffraction | | 2.6 | | 12196540 | | Colorectal cancer, Solid tumour/cancer | |
| 23 | Estrogen receptor | ESR1 | | 2IOG | X-Ray Diffraction | | 1.6 | | 17289385 | | Breast cancer, Adrenocortical carcinoma | |
| 24 | Fibroblast growth factor receptor 3 | FGFR3 | | 4K33 | X-Ray Diffraction | | 2.3 | | 23972473 | | Solid tumour/cancer | |
| 25 | Glutathione S-transferase P | GSTP1 | | 3IE3 | X-Ray Diffraction | | 1.8 | | 19808963 | | Solid tumour/cancer | |
| 26 | GTPase Hras | HRAS | | 5P21 | X-Ray Diffraction | | 1.4 | | 2196171 | | Solid tumour/cancer | |
| 27 | Guanosine Monophosphate reductase 2 | GMPR2 | | 2A7R | X-Ray Diffraction | | 3.0 | | 16359702 | | Solid tumour/cancer | |
| 28 | Heat shock protein HSP 90-alpha | HSP90AA1 | | 2QG0 | X-Ray Diffraction | | 1.9 | | 17630989 | | Multiple myeloma | |
| 29 | Interstitial collagenase | MMP1 | | 1HFC | X-Ray Diffraction | | 1.5 | | 8090713 | | Lung cancer, Brain cancer | |
| 30 | Kinesin-like protein KIF11 | KIF11 | | 1Q0B | X-Ray Diffraction | | 1.9 | | 14672662 | | Solid tumour/cancer | |
| 31 | Macrophage colony-stimulating factor 1 receptor | CSF1R | | 2I0V | X-Ray Diffraction | | 2.8 | | 17132624 | | Solid tumour/cancer, Pancreatic cancer | |
| 32 | MAP kinase-interacting serine/threonine-protein kinase 1 | MKNK1 | | 2HW6 | X-Ray Diffraction | | 2.5 | | 16917500 | | Mesothelioma | |
| 33 | MAP kinase-interacting serine/threonine-protein kinase 2 | MKNK2 | | 2HW7 | X-Ray Diffraction | | 2.7 | | 16917500 | | Mesothelioma | |
| 34 | MART-1 melanoma antigen | MLANA | | 3HG1 | X-Ray Diffraction | | 3.0 | | 19605354 | | Melanoma | |
| 35 | Matrix metallopeptidase-9 | MMP9 | | 1GKC | X-Ray Diffraction | | 2.3 | | 12051944 | | Lung cancer; Brain cancer | |
| 36 | Melanoma-associated antigen 1 | MAGEA1 | | 3BO8 | X-Ray Diffraction | | 1.8 | | 19177349 | | Lung cancer | |
| 37 | Mitogen-activated protein kinase 1 | MAPK1 | | 3SA0 | X-Ray Diffraction | | 1.6 | | 22084399 | | Solid tumour/cancer | |
| 38 | Nuclear factor erythroid 2-related factor 2 | NFE2L2 | | 3ZGC | X-Ray Diffraction | | 2.2 | | 23722832 | | Non-small-cell lung cancer | |
| 39 | Peptidyl-prolyl cis-trans isomerase NIMA-interacting 1 | PIN1 | | 1F8A | X-Ray Diffraction | | 1.8 | | 10932246 | | Non-small-cell lung cancer | |
| 40 | Peroxiredoxin-5, mitochondrial | PRDX5 | | 1H4O | X-Ray Diffraction | | 1.9 | | 11518528 | | Brain cancer | |
| 41 | Phosphatidylinositol 4,5-bisphosphate 3-kinase catalytic subunit alpha isoform | PIK3CA | | 3ZIM | X-Ray Diffraction | | 2.8 | | 23360348 | | Solid tumour/cancer,Prostate cancer | |
| 42 | Progesterone receptor | PGR | | 1A28 | X-Ray Diffraction | | 1.8 | | 9620806 | | Endometrial cancer | |
| 43 | Prolactin receptor | PRLR | | 3MZG | X-Ray Diffraction | | 2.1 | | 20889499 | | Solid tumour/cancer, Fallopian tube cancer | |
| 44 | Prostate-specific antigen | KLK3 | | 2ZCH | X-Ray Diffraction | | 2.8 | | 18187150 | | Prostate cancer | |
| 45 | Protein kinase C alpha type | PRKCA | | 4RA4 | X-Ray Diffraction | | 2.6 | | 25254961 | | Non-small-cell lung cancer | |
| 46 | Proto-oncogene tyrosine-protein kinase Src | SRC | | 1A07 | X-Ray Diffraction | | 2.2 | | 9174343 | | Solid tumour/cancer,Breast cancer | |
| 47 | Pyruvate Kinase PKM | PKM | | 6B6U | X-Ray Diffraction | | 1.4 | | 29182273 | | Renal cell carcinoma | |
| 48 | Receptor tyrosine-protein kinase erbB-2 | ERBB2 | | 2A91 | X-Ray Diffraction | | 2.5 | | 12620236 | | Breast cancer, Solid tumour/cancer | |
| 49 | Retinoic acid receptor alpha | RARA | | 3A9E | X-Ray Diffraction | | 2.7 | | 21152046 | | Solid tumour/cancer,Colorectal cancer | |
| 50 | Serine/threonine-protein kinase Chk2 | CHEK2 | | 2CN5 | X-Ray Diffraction | | 2.3 | | 16794575 | | Solid tumour/cancer | |
| 51 | Serine/threonine-protein kinase Sgk1 | SGK1 | | 2R5T | X-Ray Diffraction | | 1.9 | | 17965184 | | Prostate cancer | |
| 52 | Serine/threonine-protein phosphatase 2A catalytic subunit alpha isoform | PPP2CA | | 2IAE | X-Ray Diffraction | | 3.5 | | 17086192 | | Solid tumour/cancer | |
| 53 | Telomerase reverse transcriptase | TERT | | 2BCK | X-Ray Diffraction | | 2.8 | | 16323248 | | Brain cancer | |
| 54 | TGF-beta receptor type-II | TGFBR2 | | 1M9Z | X-Ray Diffraction | | 1.2 | | 12121646 | | Solid tumour/cancer | |
| 55 | Thymidylate synthase | TYMS | | 1HVY | X-Ray Diffraction | | 1.9 | | 11329255 | | Gastric adenocarcinoma | |
| 56 | Transforming growth factor -beta receptor type-I | TGFBR1 | | 1RW8 | X-Ray Diffraction | | 2.4 | | 15177479 | | Solid tumour/cancer | |
| 57 | Tumor necrosis factor ligand superfamily member 10 | TNFSF10 | | 1D0G | X-Ray Diffraction | | 2.4 | | 10549288 | | Brain cancer, Prostate cancer | |
| 58 | Tyrosine-protein kinase JAK2 | JAK2 | | 2B7A | X-Ray Diffraction | | 2.0 | | 16174768 | | Solid tumour/cancer | |
| 59 | Vascular endothelial growth factor receptor 1 | FLT1 | | 1RV6 | X-Ray Diffraction | | 2.5 | | 14684734 | | Colorectal cancer | |
| 60 | Vascular endothelial growth factor receptor 2 | KDR | | 2OH4 | X-Ray Diffraction | | 2.1 | | 17676829 | | Biliary cancer, Solid tumour/cancer | |
